# Trapping endoplasmic reticulum with amphiphilic AIE-active sensor via specific interaction of ATP-sensitive potassium (K_ATP_)

**DOI:** 10.1093/nsr/nwaa198

**Published:** 2020-08-31

**Authors:** Zhirong Zhu, Qi Wang, Hongze Liao, Ming Liu, Zhenxing Liu, Youheng Zhang, Wei-Hong Zhu

**Affiliations:** Shanghai Key Laboratory of Functional Materials Chemistry, Key Laboratory for Advanced Materials and Institute of Fine Chemicals, Joint International Research Laboratory of Precision Chemistry and Molecular Engineering, Feringa Nobel Prize Scientist Joint Research Center, Frontiers Science Center for Materiobiology and Dynamic Chemistry, School of Chemistry and Molecular Engineering, East China University of Science and Technology, Shanghai 200237, China; Shanghai Key Laboratory of Functional Materials Chemistry, Key Laboratory for Advanced Materials and Institute of Fine Chemicals, Joint International Research Laboratory of Precision Chemistry and Molecular Engineering, Feringa Nobel Prize Scientist Joint Research Center, Frontiers Science Center for Materiobiology and Dynamic Chemistry, School of Chemistry and Molecular Engineering, East China University of Science and Technology, Shanghai 200237, China; Research Center for Marine Drugs, State Key Laboratory of Oncogene and Related Genes, Department of Pharmacy, Ren Ji Hospital, School of Medicine, Shanghai Jiao Tong University, Shanghai 200127, China; Shanghai Key Laboratory of Functional Materials Chemistry, Key Laboratory for Advanced Materials and Institute of Fine Chemicals, Joint International Research Laboratory of Precision Chemistry and Molecular Engineering, Feringa Nobel Prize Scientist Joint Research Center, Frontiers Science Center for Materiobiology and Dynamic Chemistry, School of Chemistry and Molecular Engineering, East China University of Science and Technology, Shanghai 200237, China; Shanghai Key Laboratory of Functional Materials Chemistry, Key Laboratory for Advanced Materials and Institute of Fine Chemicals, Joint International Research Laboratory of Precision Chemistry and Molecular Engineering, Feringa Nobel Prize Scientist Joint Research Center, Frontiers Science Center for Materiobiology and Dynamic Chemistry, School of Chemistry and Molecular Engineering, East China University of Science and Technology, Shanghai 200237, China; Shanghai Key Laboratory of Functional Materials Chemistry, Key Laboratory for Advanced Materials and Institute of Fine Chemicals, Joint International Research Laboratory of Precision Chemistry and Molecular Engineering, Feringa Nobel Prize Scientist Joint Research Center, Frontiers Science Center for Materiobiology and Dynamic Chemistry, School of Chemistry and Molecular Engineering, East China University of Science and Technology, Shanghai 200237, China; Shanghai Key Laboratory of Functional Materials Chemistry, Key Laboratory for Advanced Materials and Institute of Fine Chemicals, Joint International Research Laboratory of Precision Chemistry and Molecular Engineering, Feringa Nobel Prize Scientist Joint Research Center, Frontiers Science Center for Materiobiology and Dynamic Chemistry, School of Chemistry and Molecular Engineering, East China University of Science and Technology, Shanghai 200237, China

**Keywords:** aggregation-induced emission, *amphiphilic* AIEgens, endoplasmic reticulum, targeting specificity, high-fidelity tracking

## Abstract

The current aggregation-induced emission luminogens (AIEgens) sometimes suffer from poor targeting selectivity due to undesirable aggregation in the hydrophilic biosystem with ‘*always-on*’ fluorescence or unspecific aggregation in the lipophilic organelle with prematurely activated fluorescence. Herein, we report an unprecedented ‘*amphiphilic* AIEgen’ sensor QM-SO_3_-ER based on the AIE building block of quinoline-malononitrile (QM). The introduced hydrophilic sulfonate group can well control the specific solubility in a hydrophilic system with desirable initial ‘*fluorescence-off*’ state. Moreover, the incorporated *p*-toluenesulfonamide group plays two roles: enhancing the lipophilic dispersity, and behaving as binding receptor to the adenosine triphosphate (ATP)-sensitive potassium (K_ATP_) on the endoplasmic reticulum (ER) membrane to generate the docking assay confinement effect with targetable AIE signal. The *amphiphilic* AIEgen has for the first time settled down the predicament of unexpected ‘*always-on*’ fluorescence in the aqueous system and the untargetable aggregation signal in the lipophilic organelle before binding to ER, thus successfully overcoming the bottleneck of AIEgens' targetability.

## Teaser

An unprecedented ‘*amphiphilic* AIEgen’ sensor QM-SO_3_-ER well settlesdown the predicamentof unexpected ‘*always-on*’ fluorescence in aqueous system and untargetable aggregation signalin lipophilic organelle, thus overcoming the bottleneck of AIEgen targetability.

## INTRODUCTION

**Figure 1.**
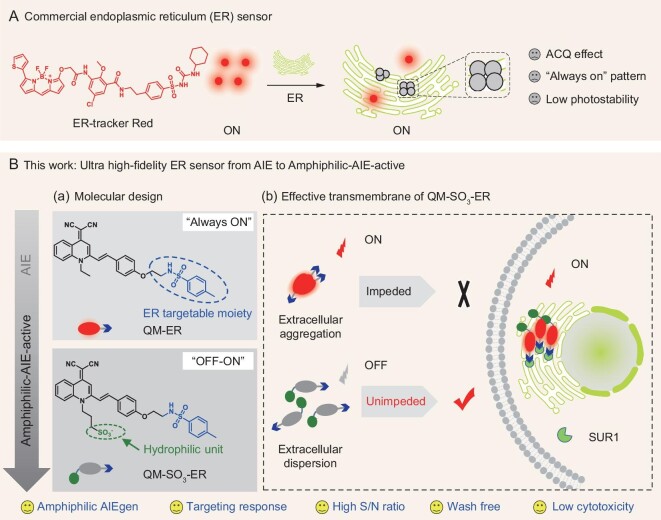
Proposing ‘*amphiphilic* AIEgen’ strategy for *in situ* high-fidelity mapping of ER. (A) Commercially-available sensor ER-tracker Red based on ACQ chromophore and fluorescence ‘*always-on*’ pattern. (B) Tracking ER with *amphiphilic* AIE-active sensor. (a) Molecular structures of QM-ER and QM-SO_3_-ER, wherein the sulfonate group is assembled into QM-ER to give QM-SO_3_-ER, and the *p*-toluenesulfonamide group serves as a moiety binding to the specific K_ATP_ on the ER membrane. (b) *Amphiphilic* QM-SO_3_-ER with superior solubility in both hydrophilic and lipophilic conditions to realize efficient cell uptake on good disperse state, thereby guaranteeing the amphiphilic AIE-active ‘*off-on*’ signal to ER enrichment through targeting group of K_ATP_ along with RIM mechanism of AIE.

The endoplasmic reticulum (ER) is an important organelle in the cell responsible for protein synthesis, transport and balance regulation of calcium ion [1,2], and ER stress is one of the prerequisites for modern immunotherapy [3]. *In situ* ER tracking and bioimaging are of increasing importance for real-time observing of dynamic intracellular processes, to take a deep insight into the pathogenesis of some metabolic diseases [4,5], like diabetes [6,7]. However, most of the commercially available ER sensors, such as ER-tracker Red and Green, are developed from aggregation-caused quenching (ACQ) fluorophores [8,9] that suffer from inherent defects such as inaccurate feedback on ER information, especially bringing up inevitable noises from ‘*always-on*’ pattern, and signal loss from poor photostability (Fig. 1A).

In contrast with ACQ fluorophore, aggregation-induced emission (AIE) sensors [10–13] bestow distinct advantages on bioimaging [13–19], especially in lighting up organelles with targeting events through the aggregation process. However, the current AIE luminogen (AIEgen) sensors are still not ideal due to the limitation of the poor targeting specificity with activated fluorescence during undesirable aggregation before AIEgens bind to the specific receptor. Given that biological research is always conducted in aqueous media, the majority of available AIE sensors are structurally modified to tune the aqueous solubility for initial disperse state, such as introducing an ionic group to the AIE building blocks, whereas most of them underscore the invalid aggregation in the lipophilic organelle [20–23].

To overcome the traditional AIE bottleneck, herein we have for the first time proposed a completely novel strategy of ‘*amphiphilic* AIEgen’ to realize good dispersity in both hydrophilic and lipophilic environments. The specific *amphiphilic* characteristic could not only prevent aggregation in an aqueous biological environment, but also keep a good disperse state once entering the lipophilic organelle to avoid false signals. It could remain in extinguished ‘*fluorescence-off*’ state during cytomembrane transport until it encounters the specific receptor action for restriction of intramolecular motion (RIM) to produce activated luminescence [10], thereby achieving the desirably selective ‘*off-on*’ fluorescence with lighting-up signal when responding to targeting events.

In the unique strategy of this *amphiphilic* AIEgen sensor (Fig. 1B), we exploited our established AIE building block of quinoline-malononitrile (QM) [24–30], and utilized the hydrophilic sulfonate group to modulate the specific solubility in the hydrophilic system with desirably initial *‘fluorescence-off’* state. Furthermore, the incorporated *p*-toluenesulfonamide group could play dual roles for realizing the *amphiphilic* characteristic, such as enhancing the lipophilic dispersity and behaving as binding receptor to the ATP-sensitive potassium (K_ATP_) on the ER membrane. By virtue of harnessing this strategy, the elaborated *amphiphilic* AIEgen sensor QM-SO_3_-ER exhibits superior dispersity in both hydrophilic and lipophilic systems with initial ‘*fluorescence-off*’ state when compared with the lipophilic QM-ER that aggregates tightly in a hydrophilic system with *‘always-on’* fluorescence (Fig. 1B). Particularly, the docking assay of QM-SO_3_-ER with K_ATP_ channel protein, wherein the subunit of sulfonylurea receptor 1 (SUR1) locates on ER, can further address the targeting mechanism.

The *amphiphilic* AIEgen sensor QM-SO_3_-ER always keeps good dispersity in either the hydrophilic or lipophilic system, thereby strongly eliminating the background fluorescence from unexpected AIE signals caused by uncontrollable polarity change. The targeting interaction between QM-SO_3_-ER and K_ATP_ can exert the specific responsiveness to ER through molecular accumulation along with the AIE lighting-up signal based on RIM mechanism (Fig. 1B). In addition, the fluorescence ‘*off-on*’ *amphiphilic* AIE-active property of QM-SO_3_-ER can facilitate the cell staining procedure with wash-free property and good photostability for long-time imaging. To the best of our knowledge, this is the first report about the *amphiphilic* AIE-active sensor, with excellent targeting ability in overcoming the bottleneck of traditional AIE fluorophore, expanding the promising toolbox used to achieve high targeting response via the interaction of AIEgens and specific protein for *in situ* and *in vivo* tracking.

## RESULTS AND DISCUSSION

### Revealing *amphiphilicity* of AIEgen sensor with synergetic interaction of sulfonate and sulfonamide groups

AIE sensors bestow distinct advantages [31–33] in lighting up organelles through the aggregation process. However, previously, most AIEgens could only disperse well in either hydrophilic or lipophilic systems [34,35] that always lead to uncontrollable molecular aggregation in the complicated physiological environment. To keep good dispersity in both hydrophilic and lipophilic environments, we propose a novel and ideal strategy called ‘*amphiphilic* AIEgen’ to solve the traditional AIE bottleneck, that is, avoiding undesirable aggregations with the ‘*fluorescence-off*’ state during cytomembrane and organelle transport. Previously, our group has reported a novel AIE building block of QM, which replaces the oxygen in the traditional dicyanomethylene-4*H*-pyran (DCM) chromophore. The AIEgen derivatives of QM have been broadly explored for fluorescent sensors, bioimaging agents, optical waveguides and drug delivery applications [24–30]. In this work, the design strategy of an ideal QM-based *amphiphilic* AIEgen is depicted in Fig. 1.

Firstly, the AIE building block of QM was utilized as a core structure to overcome the enrichment quenching effect, then the π-conjugated backbone was used to extend the long emission wavelength, and the hydroxyl group was introduced for further modification to afford the intermediate QM-OH (Supplementary Scheme S1). However, as a typical AIEgen biosensor, QM-OH prefers to aggregate as AIE dye aggregates in an aqueous environment, and causes a ‘*false*’ positive signal with low signal/noise (*S*/*N*) from fluorescence ‘*always on*’ states. Subsequently, the ethyl group of QM-OH was replaced with a hydrophilic propylsulfonate group to obtain QM-SO_3_-OH, which could well control the specific solubility in the hydrophilic system with desirably initial ‘*fluorescence-off*’ state (Supplementary Fig. S1). Finally, the *p*-toluenesulfonamide group was grafted on the hydroxyl group of QM-SO_3_-OH by ethyl linker to give the desirable sensor QM-SO_3_-ER. It is anticipated that the incorporated sulfonamide group could play two roles in the *amphiphilic* AIEgen, that is, enhancing the lipophilic dispersity and behaving as the specifically binding receptor to K_ATP_ on the ER membrane. In this regard, it is the synergetic interaction of both the hydrophilic sulfonate group and the binding receptor of sulfonamide group that can result in the desirable *amphiphilicity* with AIE-active sensor. The chemical structures of QM-SO_3_-OH and intermediates were well confirmed by ^1^H and ^13^C nuclear magnetic resonance (NMR), and high-resolution mass spectroscopy (HRMS) in the Supplementary Data.

### Minimizing undesirable aggregation with superior dispersity of *amphiphilic* QM-SO_3_-ER

There exist two major obstacles in the reported AIEgen' sensors: (i) the tight aggregation in the aqueous biosystem with ‘*always-on*’ fluorescence, and (ii) the improper unexpected aggregation in the lipophilic organelle with activated fluorescence. Here the specific *amphiphilicity* of AIE-active QM-SO_3_-ER is anticipated to eliminate the undesirable fluorescence lighting-up with superior dispersity or solubility in both aqueous and lipophilic systems before binding to the targeting receptor, thereby overcoming the bottleneck of AIEgens' targetability.

**Figure 2.**
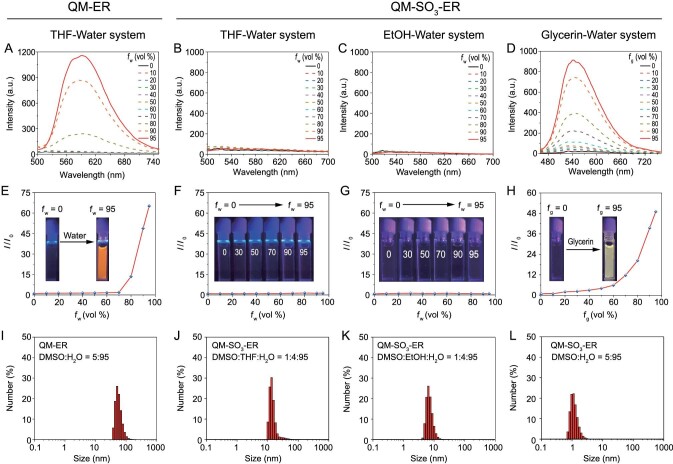
AIE properties of QM-ER and QM-SO_3_-ER with different water fractions (*f*_w_) in various solvents. (A) Emission spectra of QM-ER (10 μM) in a mixture of THF-water system (*λ*_ex_ = 447 nm). (B–D) Emission spectra of QM-SO_3_-ER (10 μM) in different solvents-water systems (*λ*_ex_ = 447 nm). (E) *I*/*I*_0_ plots of QM-ER and (F–H) QM-SO_3_-ER. *I* is the fluorescence intensity of fluorophore in 95% water at 589 nm for (E–G), and 95% glycerin at 540 nm for (H); *I*_0_ is the fluorescence intensity of fluorophore in 0% water for (E–G), and 0% glycerin for (H). (I) Hydrodynamic diameter of QM-ER (10 μM) in a mixture of DMSO/water (v/v = 5 : 95), and (J–L) Hydrodynamic diameter of QM-SO_3_-ER (10 μM) in a mixture of DMSO : THF : H_2_O (v/v/v = 1 : 4 : 95), DMSO : EtOH/H_2_O (v/v/v = 1 : 4 : 95) and DMSO/H_2_O (v/v = 5 : 95), respectively, obtained from dynamic light scattering (DLS).

The AIE properties of QM-ER were evaluated in tetrahydrofuran (THF)-water mixtures with different fractions of water (*f*_w_). In contrast to the ACQ characteristics of commercial sensors such as ER-tracker Red (Supplementary Fig. S2), QM-ER possesses the classical AIE characteristic of solving the engaged quenching problem. Specifically, the emission of QM-ER became increased quickly and monotonously when the *f*_w_ was higher than 70%, yielding a luminous orange signal with an emission peak at 589 nm (Fig. 2A and E). The resulting fluorescence is a typical AIE behavior, which is highly relative to the formation of QM-ER aggregates with RIM mechanism in a water environment [10,27,36]. It was further confirmed by dynamic light scattering (DLS, Fig. 2I) and transmission electron microscopy (TEM, Supplementary Fig. S3A). Although QM-ER has solved the ACQ problem, the poor aqueous solubility limits its further application in aqueous biological imaging.

To validate our proposal that the *amphiphilic* AIEgen QM-SO_3_-ER could generate a non-susceptible initial ‘*fluorescence-off*’ signal, a series of spectral properties were conducted. As expected, QM-SO_3_-ER did not emit any fluorescence signal in THF-water (Fig. 2B and F), ethanol (EtOH)-water (Fig. 2C and G) and DMSO-water system (Supplementary Figs S4 and S5) at any water fractions, or in the liposomes (Supplementary Fig. S6), suggestive of the specific *amphiphilicity* of QM-SO_3_-ER with free intramolecular motions in good disperse state [10]. As direct evidence, the results of TEM (Supplementary Fig. S3B–D) and DLS (Fig. 2J–L and Supplementary Fig. S7) indicated that QM-SO_3_-ER dispersed well in water phase with undetectable hydrodynamic diameter. Moreover, the Partition-coefficient (Log*P*_o/w_) of QM-SO_3_-ER (1.61) is bigger than QM-ER (1.23), suggesting better hydrophilicity. The unique *amphiphilicity* could be ascribed to the synergetic contribution: (i) the hydrophilic sulfonate group increases the aqueous solubility, and (ii) the grafted *p*-toluenesulfonamide group enhances the dispersity in the lipophilic system [26,37].

Furthermore, the intrinsic AIE behavior of QM-SO_3_-ER was demonstrated with increasing viscosity, that is, upon increasing the fraction of glycerin (*f*_g_); the increasingly enhanced viscosity in the glycerin-water system can recover the lighting-up AIE behavior of QM-based AIEgen via eliminating the non-radiative channel with the specific RIM mechanism [24,27]. In the high viscosity system, the free motion of QM-SO_3_-ER is restricted to release the excited state energy as a form of radiative transition. Specifically, the fluorescence intensity of QM-SO_3_-ER at *f*_g_ = 95% could see a 49-fold increase on its initial intensity (Fig. 2D and H), which exactly corresponds to the solid state fluorescence (Supplementary Fig. S8). Here we expect that the *amphiphilicity* of QM-SO_3_-ER might make an innovative breakthrough that keeps good disperse state in a wide range of hydrophilic-lipophilic environments [38]. It is highly desirable that the *amphiphilic* QM-SO_3_-ER could avoid undesirable aggregation with the *‘fluorescence-off’* state during cytomembrane transport until it encounters the specific receptor action to restrict the intramolecular motion with the specific ‘*off-on*’ activatable fluorescence response, thus possibly achieving the high mapping feedback and overcoming the bottleneck to AIEgens' targetability.

### *Amphiphilic* AIEgen: targeting mechanism with molecular docking

**Figure 3.**
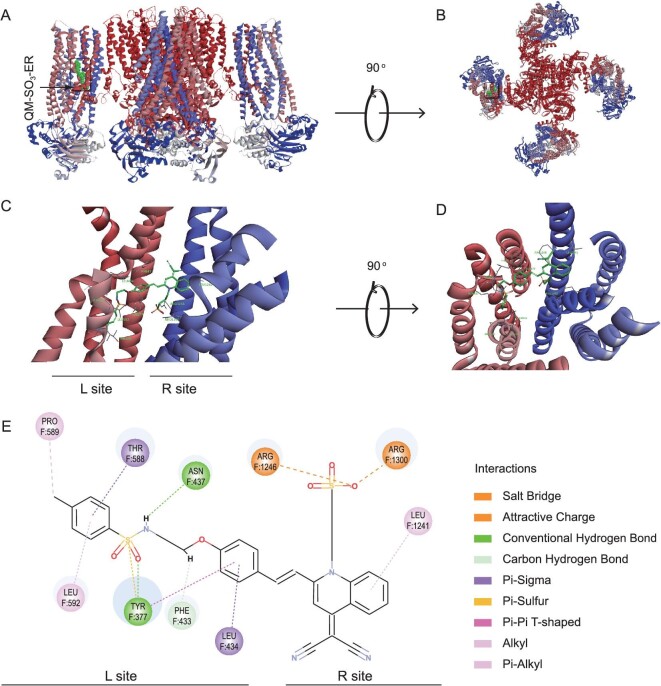
Molecular docking of QM-SO_3_-ER with K_ATP_ channel. (A) Structural model of the K_ATP_ channel binding to QM-SO_3_-ER in boxes viewed from side position. (B) View of the model from the extracellular side. (C and D) Close-up of QM-SO_3_-ER binding site in SUR1. (E) The specific interactions between QM-SO_3_-ER and SUR1. PDB ID of K_ATP_ is 6BAA.

The SUR1 domain (subunit of K_ATP_ channel located on ER membrane) is the typical binding site of sulfonamide moiety [39], wherein the commercial ER-tracker Red could bind to the ER membrane with the assistance of glibenclamide (a drug for type 2 diabetes). Hence, we envisaged that sensor QM-SO_3_-ER might have a similar interaction with an ER organelle. To figure out the targeting mechanism of QM-SO_3_-ER to K_ATP_ channel protein, the molecular docking assay was exploited to gain insight into the intrinsic binding sites (Supplementary Fig. S9). As expected, sensor QM-SO_3_-ER could specifically bind to the SUR1 with a similar mode to glibenclamide (Fig. 3A). Specifically, the ER-targeting sulfonamide unit in sensor QM-SO_3_-ER intimately interacts with the residues in the pocket of TMD0 in SUR1 domain, wherein the residues F589, F588, F437, F434, F433, F337 and F592 can form a well-tailored pocket to accommodate the targeting moiety and conjugated benzene ring. The conventional hydrogen bond, *π*–*σ*, alkyl and *π*-alkyl are the predominant interactions assisting the docking position. The binding site F433 is found to overlap with that of glibenclamide, which further confirms the irreplaceable role of ER-targeting moiety. Surprisingly, another hydrophilic sulfonate group also has interaction with this binding site through the Salt Bridge and Attractive Charge, thus resulting in a stronger interaction with high bonding affinity. Furthermore, the AIE building block of QM could also interact with residues F1241 through *π*-alkyl interaction.

The molecular docking study results indicate that both sulfonamide and the sulfonate group could bind to SUR1 domain specifically. In this regard, the targeting interaction between QM-SO_3_-ER and SUR1 domain (subunit of K_ATP_ channel located on the ER membrane) ensures the specific responsiveness to ER from two aspects that—confinement effect being caused by SUR1 domain as confinement-induced emission (CIE) and targeting-caused molecular aggregation with AIE emission—thereby result in the docking assay on the restricted intramolecular motion in limited space. As a consequence, the ER-tracking process could be proposed as follows: the *amphiphilic* AIEgen QM-SO_3_-ER crosses through the cell membrane unimpededly, travels in the cytoplasm without any unexpected fluorescence owing to the good dispersity or solubility with unique *amphiphilicity*, then targets to SUR1 on the ER membrane with the assistance of ER-targeting moiety (Fig. 1B). Followed by the enrichment in ER, the intramolecular motion is restricted from the docking assay with the interaction between QM-SO_3_-ER and the subunit of the K_ATP_ channel located on the ER membrane, thereby anticipating the achievement of a targetable response with active fluorescence.

### Improving transmembrane efficiency and ER-targeting ability

**Figure 4.**
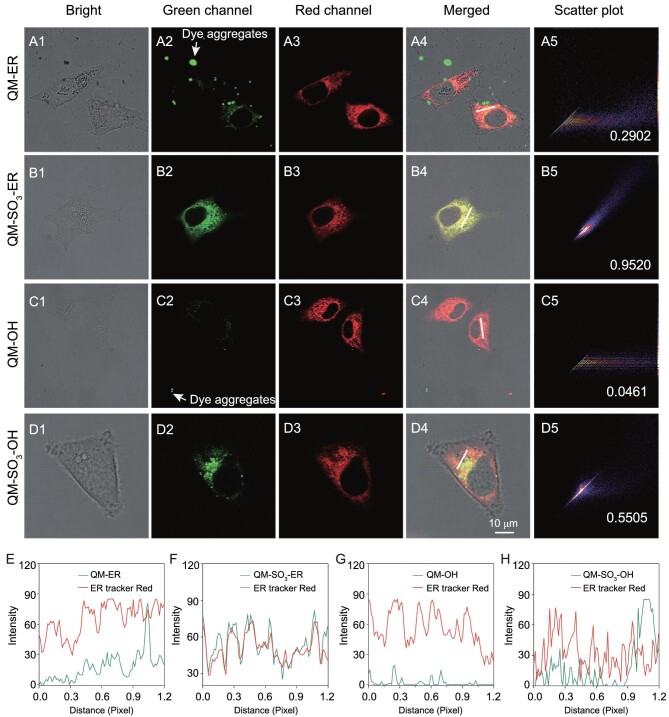
Efficient transmembrane and excellent ER-targeting ability of QM-SO_3_-ER with HeLa cells. (A1–D1) Bright channels incubated with QM-ER, QM-SO_3_-ER, QM-OH and QM-SO_3_-OH using a concentration of 3 μM for 2 h, and followed by co-staining with ER-tracker Red (1 μM) for 30 min. (A2–D2) Green channels obtained from QM-ER, QM-SO_3_-ER, QM-OH and QM-SO_3_-OH (*λ*_ex_ = 405 nm, *λ*_em_ = 550–630 nm). (A3–D3) Red channels obtained from ER-tracker Red (*λ*_ex_ = 561 nm, *λ*_em_ = 580–630 nm). (A4–D4) Merging of green, red and bright channels. (A5–D5) Intensity scatter plots of QM-ER, QM-SO_3_-ER, QM-OH and QM-SO_3_-OH with ER-tracker Red, respectively. Insert: Pearson's correlation coefficient. (E–H) The intensity profile of the linear region of interest (ROI) across the cells in A4–D4.

The transmembrane abilities of QM-OH, QM-ER, QM-SO_3_-OH and QM-SO_3_-ER were evaluated by incubating them with HeLa cells, and comparing them to a commercial ER-specific fluorescent dye (ER-tracker Red, Fig. 1). As shown in Fig. 4C, the water-insoluble fluorophore QM-OH was mostly detained in extracellular matrix even when washing with phosphate buffer saline (PBS) three times, along with ‘*always on*’ fluorescence background interference. It was suggestive that QM-OH might predominantly accumulate outside HeLa cells as AIE dye aggregates, in exact line with the aforementioned spectral property (Supplementary Fig. S1). Its poor Pearson's correlation coefficient (0.0461) also reflected the undesirable co-localization result (Fig. 4C). For another water-insoluble QM-ER, the poor penetration efficiency was also observed with a similar low Pearson's correlation coefficient (0.2902, Fig. 4A). In contrast, the hydrophilic QM-SO_3_-OH (Fig. 4D) and *amphiphilic* QM-SO_3_-ER (Fig. 4B) can easily penetrate the cell membrane owing to the dispersive molecular state in the aqueous bio-environment. Here, the transmembrane ability and targeting effect are the obvious preconditions to map ER accurately. However, most traditional AIE sensors tend to aggregate in an aqueous biological system [21,22] during the transmembrane process because of the poor solubility in a hydrophilic system, such as QM-OH and QM-ER.

For achieving the specific targeting ability, the *amphiphilicity of* AIEgen sensor QM-SO_3_-ER is expected to ensure cellular internalization owing to the good dispersity in hydrophilic and lipophilic systems. As demonstrated with reference to ER-tracker Red in HeLa cells, the *amphiphilic* QM-SO_3_-ER was co-localized very well with high Pearson's correlation coefficient of 0.9520 (Fig. 4B), indicative of highly desirable ER-targeting ability. However, the hydrophilic QM-SO_3_-OH exhibited a much poorer Pearson's correlation coefficient of 0.5505 (Fig. 4D). The ER-targeting ability of QM-SO_3_-ER was further confirmed with high Pearson's correlation coefficient (Supplementary Fig. S10) in pancreatic cancer cells (PANC-1), human adenocarcinoma cell (A549) and human hepatocellular carcinoma cell (7701). Benefiting from the molecular dispersion in aqueous and lipophilic systems, the *amphiphilicity* guarantees that the AIE-active sensor of QM-SO_3_-ER can not only get across the cell membrane with high internalization ability and inactive ‘*fluorescence-off*’ signal, but also enhance the lipophilic dispersity to behave as binding receptor to K_ATP_ on the ER membrane (Fig. 3), wherein the AIE lighting-up response can be activated on the base of the RIM mechanism through the specific confinement effect of molecular docking [10]. In this regard, it is the first time the predicament of the unexpected ‘*always-on*’ fluorescence signal from undesirable aggregation in an aqueous biology system and the unexpected aggregation signal in a lipophilic organelle before binding to ER has been settled down, thus making a breakthrough in achieving the high mapping feedback and overcoming the bottleneck to AIEgens' targetability.

### Wash-free behavior and ER trapping with ultra-high *S/N* ratio

**Figure 5.**
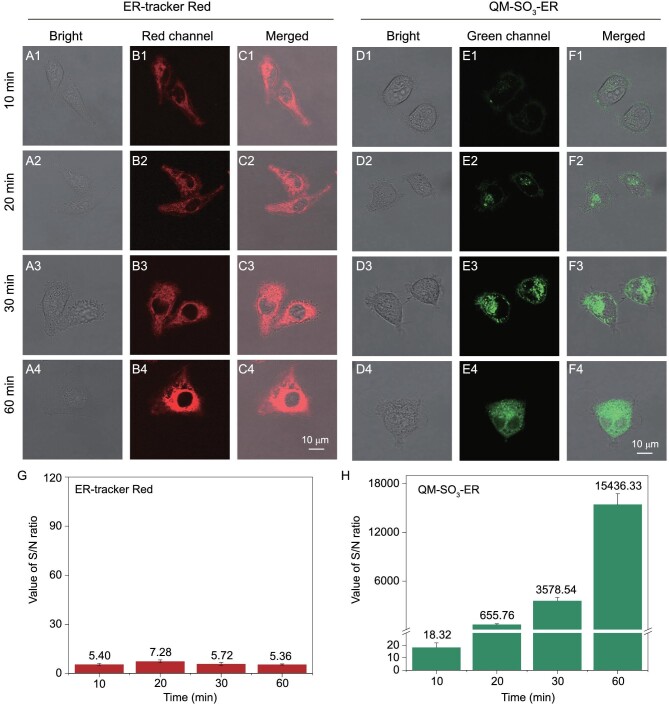
Non-fluorescence in the aqueous system of QM-SO_3_-ER guaranteeing the wash-free behavior for ER imaging. HeLa cells incubated with (A–C) ER-tracker Red (1 μM) and (D–F) QM-SO_3_-ER (3 μM) for different times, green channels obtained from QM-SO_3_-ER (*λ*_ex_ = 405 nm, *λ*_em_ = 550–630 nm), and red channels obtained from ER-tracker Red (*λ*_ex_ = 561 nm, *λ*_em_ = 580–630 nm). (G and H) The *S*/*N* ratios of ER-tracker Red and QM-SO_3_-ER at different times. Note: sensor QM-SO_3_-ER is *lighting-up* feature with low background (F1–F4), while ER-tracker is fluorescence ‘*always-on*’ feature with obvious background around the HeLa cells (C1–C4). The *S*/*N* value of QM-SO_3_-ER increases with the incubating time in contrast with that of ER-tracker Red.

The ER staining images of HeLa cells were further recorded with QM-SO_3_-ER upon incubating with ER-tracker Red and QM-SO_3_-ER for 10, 20, 30 and 60 min, respectively. Obviously, ER-tracker Red could enter cells and stain on the ER within 10 min, but the obvious background interference around the cells interrupted the merged images (Fig. 5B and C). In contrast, QM-SO_3_-ER could gradually enter the cell in a time dependent manner without any extracellular background fluorescence, and gave more clear information in the merged images (Fig. 5E and F) with wash-free behavior. It took 30–60 min to light up the whole ER part with QM-SO_3_-ER due to the AIEgen RIM effect with molecular docking confinement. Moreover, the *S*/*N* ratio of *amphiphilic* QM-SO_3_-ER increased from 18.32 (10 min) to 15 436.33 (60 min, Fig. 5H), much larger than the commercially available ER-tracker Red (in the range between 5.36 and 7.28) in the same time period (Fig. 5H). Indeed, the *amphiphilic* AIEgen QM-SO_3_-ER is indicative of the minimal background interference and ultra-high sensitivity, especially for minimal background interference and ultra-high *S*/*N* ratio from both free dye and bio-substrate auto-fluorescence [34,40–43].

### Long-time tracking with excellent intra- and extra-cellular photostability

**Figure 6.**
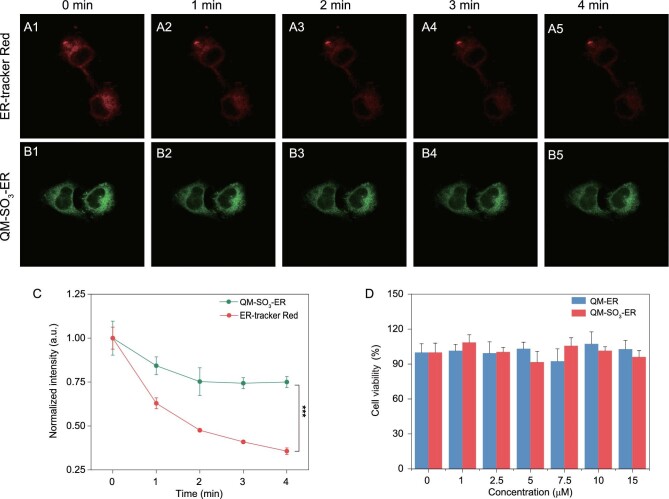
Excellent intracellular photostability of QM-SO_3_-ER. (A1–A5) Confocal images of HeLa cells stained with ER-tracker Red (*λ*_ex_ = 561 nm, *λ*_em_ = 580–630 nm) and (B1–B5) QM-SO_3_-ER (*λ*_ex_ = 405 nm, *λ*_em_ = 550–630 nm) at different scan times. (C) Normalized fluorescence signal loss of QM-SO_3_-ER and ER-tracker Red. (D) 3-(4,5-Dimethylthiazol-2-yl)-2,5-diphenyltetrazolium bromide (MTT) assays of QM-ER and QM-SO_3_-ER at different concentrations. Note: the cell viability of QM-ER and QM-SO_3_-ER is not obviously different when compared with the control group (n = 3, data expressed as average ± standard error; statistical significance: *P* values, ^*^ represents *P* < 0.05, ^*^^*^ represents *P* < 0.01 and ^*^^*^^*^ represents *P* < 0.001, calculated with the Student's T-test).

Photostability is an important parameter for bioluminescent imaging agents [37,44–47]. In particular, there exists serious photobleaching and image distortion especially for multiple tracking acquisition cycles and prolonged light exposure of sensors. Structurally, the bonding force of fluoroboron coordination bonds in ER-tracker Red is much weaker than covalent bonds in QM-SO_3_-ER, thus generating poor photostability. Here we compared the photostability of ER-tracker Red, QM-ER and QM-SO_3_-ER upon continuous light irradiation for 20 minutes. As shown in the time-dependent absorbance, the *A*/*A*_0_ values of QM-ER and QM-SO_3_-ER remained above 80% while that of ER-tracker Red fell down below 50% (Supplementary Fig. S11). Also, the photostabilities of QM-SO_3_-ER and ER-tracker Red were conducted in intra-cellular environments. After sequentially scanning for 4 min in living HeLa cells, to the naked eye, the fluorescent signal of QM-SO_3_-ER decreased slightly from 0 to 2 min and then kept stable (Fig. 6B), which is in line with the normalized intensity (Fig. 6C). However, ER-tracker Red exhibited poorer behavior, and decreased the signal sharply until it was almost invisible (Fig. 6A) with about 70% signal loss (Fig. 6C). Obviously, QM-SO_3_-ER exhibits better photostability than commercial sensor ER-tracker Red. In the meantime, upon incubating HeLa cells, *amphiphilic* AIEgen QM-SO_3_-ER showed similar cell viability when compared with a control group (fresh Dulbecco's modified eagle medium (DMEM)) after 24 h, suggestive of negligible toxicity during the ER trapping process (Fig. 6D). The HeLa cells maintained good cell viability at the concentration of 15 μM, which was five times higher than that used for cell imaging study. Taken together, QM-SO_3_-ER exhibits long-time ER bioimaging with excellent photostability and low toxicity, serving as an alternative to the commercially available sensor ER-tracker Red.

## CONCLUSION

We have for the first time proposed the unique strategy of ‘*amphiphilic* AIEgen’ with good solubility and dispersity in both hydrophilic and lipophilic systems, for the sake of solving the traditional AIE bottleneck of poor specific targeting with *in vivo* high-fidelity trapping of ER. Compared to the commercially available ER-tracker Red, the unprecedented *amphiphilic* AIEgen QM-SO_3_-ER exhibited superior targeting capability from three aspects: (i) the AIE building block QM could overcome the ACQ effect with enhanced photostability; (ii) the grafted sulfonate group could well control the specific solubility in a hydrophilic system for initial ‘*fluorescence-off*’ state, and the incorporated *p*-toluenesulfonamide group increased the lipophilic dispersity to avoid untargetable aggregation in the organelle; and (iii) the specific *amphiphilicity* could guarantee superior solubility or dispersion in both hydrophilic and lipophilic systems, thereby achieving superior targeting through binding receptor to K_ATP_ on the ER membrane to generate the docking assay confinement effect on the restriction of intramolecular motion, along with recovering the specific AIE-active lighting-up fluorescence signal. It is the specific *amphiphilicity* of QM-SO_3_-ER that solves the predicament of the unexpected ‘*always-on*’ fluorescence signal from undesirable aggregation in a hydrophilic bioimaging system and the unexpected aggregation signal in a cell organelle before binding to ER, and strongly eliminates the background fluorescence from unexpected AIE signals caused by uncontrollable polarity change, thereby achieving high-fidelity mapping feedback and overcoming the bottleneck to AIEgens' targetability. Both the cell co-localization experiment and docking study provide evidence on the accurate feedback of *in situ* mapping of the ER with extraordinary features, such as beneficial wash-free behavior, ultra-high time-dependent *S*/*N* sensitivity, as well as high intrinsic photostability and low cytotoxicity. With regard to the ACQ effect and the fluorescence *‘always on’* pattern of the commercial ER-tracker Red, the AIE-active sensor with the *amphiphilic* strategy can pave a novel and straightforward pathway to building up a high-fidelity AIE trapping sensor, avoiding a false signal from undesirable aggregation before binding to the specific receptor, and making a breakthrough in overcoming the traditional AIE bottleneck to targeting capability, along with high selectivity via the specific receptor interaction.

## Supplementary Material

nwaa198_Supplemental_File
