# Supplementary material for: Trapping endoplasmic reticulum with amphiphilic AIE-active sensor via specific interaction of ATP-sensitive potassium (KATP)
Source: Natl Sci Rev. 2020 Aug 31;8(6):nwaa198. doi: 10.1093/nsr/nwaa198 (PMC8288166; doi:10.1093/nsr/nwaa198)
Supplement: nwaa198_Supplemental_File [file nwaa198_supplemental_file.docx]

**Trapping endoplasmic reticulum with amphiphilic AIE-active sensor *via* specific interaction of ATP-sensitive potassium (K_ATP_)**

Zhirong Zhu^1^, Qi Wang^1,^*, Hongze Liao^2^, Ming Liu^1^, Zhenxing Liu^1^, Youheng Zhang^1^ and Wei-Hong Zhu^1,^*

*^1^Shanghai Key Laboratory of Functional Materials Chemistry, Key Laboratory for Advanced Materials and Institute of Fine Chemicals, Joint International Research Laboratory of Precision Chemistry and Molecular Engineering, Feringa Nobel Prize Scientist Joint Research Center, Frontiers Science Center for Materiobiology and Dynamic Chemistry, School of Chemistry and Molecular Engineering, East China University of Science and Technology, Shanghai 200237, China.*

*^2^Research Center for Marine Drugs, State Key Laboratory of Oncogene and Related Genes, Department of Pharmacy, Ren Ji Hospital, School of Medicine, Shanghai Jiao Tong University, Shanghai 200127, China*

***Corresponding authors**. E-mail: [whzhu@ecust.edu.cn](mailto:whzhu@ecust.edu.cn); [wangqi@ecust.edu.cn](mailto:wangqi@ecust.edu.cn)

Contents

[1. Experimental section 3](#_Toc35952276)

[2. Absorption and emission spectra of QM-OH and QM-SO_3_-OH 7](#_Toc35952277)

[3. ACQ effect of ER-tracker Red 8](#_Toc35952278)

[4. TEM images of QM-ER and QM-SO_3_-ER 8](#_Toc35952279)

[5. Absorption spectra of QM-ER and QM-SO_3_-ER in various systems 8](#_Toc35952280)

[6. Absorption and emission spectra of QM-SO_3_-ER in DMSO-water systems 9](#_Toc35952281)

[7. Emission spectra of QM-SO_3_-ER in liposomes 9](#_Toc35952282)

[8. Hydrodynamic diameter of QM-SO_3_-ER 10](#_Toc35952283)

[9.](#_Toc35952284) [Solid state fluorescence spectra of QM-ER and QM-SO_3_-ER 11](#_Toc35952285)

[10. Overall structure of the K_ATP_ channel bound QM-SO_3_-ER 11](#_Toc35952286)

[11. Co-localization experiment of QM-SO_3_-ER and ER-tracker Red 12](#_Toc35952287)

[12. Photostability of ER-tracker Red, QM-ER and QM-SO_3_-ER *in vitro* 12](#_Toc35952288)

[13. Characterization of compounds 13](#_Toc35952289)

1. Experimental section

1.1 Materials and instruments

All solvents and chemicals, unless special stated, were purchased commercially in analytical grade and used without further purification. ^1^H and ^13^C NMR spectra in deuterium generation reagent were obtained with a Bruker AvanceIII 400 MHz NMR spectrometer using TMS as an internal standard. High resolution mass spectrometry (HRMS) spectra were measured with a Waters LCT Premier XE spectrometer. UV-Vis absorption and fluorescence spectra were recorded on an Agilent Cary 60 spectrophotometer and Varian Cary Eclipse fluorescence spectrophotometer, respectively (10 × 10 mm quartz cuvette). Dynamic light scatting (DLS) experiments were conducted with Zetasizer Nano-ZS (Malvern Instruments, Worcestershire, UK). TEM images on a JEOL JEM-1400 and Hitachi H-9500 transmission electron microscope. Confocal fluorescence images were performed on confocal laser scanning microscope (CLSM, Leica confocal microscope TCS SPS CFSMP).

1.2 Synthesis of compounds QM-ER and QM-SO_3_-ER

**Scheme S1.** Synthetic route of compounds QM-ER and QM-SO_3_-ER.

1.3 Synthesis of ER-targeting moiety

2-Chloroethylamine hydrochloride (348.0 mg, 3.0 mmol) and *p*-toluenesulfonyl chloride (525.0 mg, 3.0 mmol) were dissolved in dry DMF (5 mL) under nitrogen protection at room temperature in a 50 mL dried three-necked flask. 2 mL of triethylamine was added dropwise under the ice bath agitation, and keep stirring for 3 h. The mixture was poured into a cold solution of 5% HCl (150.0 mL) for filtration and followed by drying to get white solid powder (645.9 mg): yield 90.21%. ^1^H NMR (400 MHz, DMSO-*d*_6_, ppm): *δ* 7.92 (d, *J* = 4.8 Hz, 1H, N-H), 7.69 (d, *J* = 8.4 Hz, 2H, Ph-H), 7.41 (d, *J* = 8.0 Hz, 2H, Ph-H), 3.56 (t, *J* = 6.4 Hz, 2H, ClC*H_2_*CH_2_), 3.04 (q, *J* = 12.0, 6.0 Hz, ClCH_2_C*H_2_*), 2.39 (s, 3H, CH_3_). ^13^C NMR (100 MHz, DMSO-*d*_6_, ppm): *δ* 20.93, 43.41, 44.34, 126.46, 129.64, 137.53, 142.76. High-resolution mass spectrometry (ESI positive ion mode for [M + H]^+^): Calcd. for C_9_H_12_NO_2_SCl: 256.0175; found: 256.0165.

1.4 Synthesis of QM-OH

QM (1.0 g, 4.3 mmol) and 4-hydroxybenzaldehyde (1.7 g, 13.8 mmol) were dissolved in acetonitrile (50.0 mL) with piperidine (1.0 mL) under argon protection at room temperature. The mixture was then refluxed for 10 h. The solvent was removed by filtration, and the crude product was purified by recrystallization to afford the desired product QM-OH (348.0 mg): yield 24.31%.^1^H NMR (400 MHz, DMSO-*d*_6_, ppm): *δ* 10.00 (s, 1H, -OH), 8.92 (q, *J* = 8.4 Hz, 1H, Ph-H), 8.02 (d, *J* = 9.2 Hz, 1H, Ph-H), 7.91 (t, *J* = 7.6 Hz, 1H, Ph-H), 7.67 (d, *J* = 8.8 Hz, 2H, Ph-H), 7.61 (t, *J* = 7.6 Hz, 1H, Ph-H), 7.37 (d, *J* = 16.0 Hz, 1H, alkene-H), 7.36 (d, *J* = 15.6 Hz, 1H, alkene-H), 7.01 (s, 1H, C=C*H*), 6.84 (d, *J* = 8.8 Hz, 2H, Ph-H), 4.57 (q, *J* = 6.8 Hz, 2H, C*H*_2_CH_3_), 1.40 (t, *J* = 6.8 Hz, 3H, CH_2_C*H*_3_). ^13^C NMR (100 MHz, DMSO-*d_6_*, ppm): *δ* 13.66, 43.78, 46.16, 48.51, 106.40, 115.78, 116.73, 118.13, 120.54, 124.84, 124.99, 126.10, 130.01, 130.62, 133.62, 137.77, 140.01, 149.65, 151.99. High-resolution mass spectrometry (ESI positive ion mode for [M + H]^+^): Calcd. for C_22_H_18_N_3_O: 340.1450; found: 340.1441.

1.5 Synthesis of QM-ER

QM-OH (270.0 mg, 0.8 mmol) and *N*-(2-chloroethyl)-4-methylbenzenesulfonamide (186.4 mg, 0.8 mmol) were dissolved in DMF (5.0 mL) with K_2_CO_3_ (55.2 mg 0.4 mmol), under argon protection at room temperature, followed by heating at 45 °C for 12 hours. The reaction solution was gradually poured into the 5% HCl solution (100.0 mL) under stirring to precipitate a large amount of red solid, which was then filtered with a funnel. The residue was separated by silica gel column chromatography to obtain the compound QM-ER (45.0 mg): yield: 10.49%. ^1^H NMR (400 MHz, DMSO-*d****_6_***, ppm): *δ* 8.93 (d, *J* = 8.4 Hz, 1H, Ph-H), 8.09 (d, *J* = 9.2 Hz, 1H, N-H), 7.91 (m, 2H, Ph-H), 7.76 (d, *J* = 8.4 Hz, 1H, Ph-H), 7.71 (d, *J* = 8.0 Hz, 1H, Ph-H), 7.61 (t, *J* = 8.0 Hz, 1H, Ph-H), 7.38 (m, 4H, 2H for alkene-H, another 2H for Ph-H), 7.01 (s, 1H, C=CH), 6.93 (d, *J* = 8.8 Hz, 2H, Ph-H), 4.57 (q, *J* = 13.6, 6.8 Hz, 2H, C*H_2_*CH_3_), 4.02 (t, *J* = 5.6 Hz, 2H, OC*H_2_*CH_2_), 3.14 (q, *J* = 11.2, 5.6 Hz, 2H, OCH_2_C*H_2_*), 2.38 (s, 1H, Ph-C*H_3_*), 1.40 (t, *J* = 6.8 Hz, 3H, CH_2_C*H_3_*). ^13^C NMR (100 MHz, DMSO-*d_6_*, ppm): *δ* 13.64, 20.94, 41.86, 43.78, 46.63, 66.50, 106.59, 114.70, 118.00, 118.08, 120.55, 124.83, 125.04, 126.49, 127.97, 129.58, 129.74, 133.59, 137.60, 137.74, 139.37, 142.61, 149.33, 152.07, 159.53. High-resolution mass spectrometry (ESI positive ion mode for [M + Na]^+^): m/z: Calcd. for C_31_H_28_N_4_O_32_SNa: 559.1780; found: 559.1783.

1.6 Synthesis of QM-SO_3_-OH

3-(4-(dicyanomethylene)-2-methylquinolin-1(4*H*)-yl) propane-1-sulfonate (QM-SO_3_, 750.0 mg, 2.3 mmol) and 4-hydroxybenzaldehyde (738.0 mg, 5.7 mmol) were dissolved in acetonitrile (50.0 mL) with piperidine (1.0 mL) under argon protection at room temperature, followed by refluxing for 10 h. The solvent was removed by filtration, and the crude product was purified by recrystallization to afford the desired product QM-SO_3_-OH (343.3 mg): yield 34.81%. ^1^H NMR (400 MHz, DMSO-*d*_6_, ppm): *δ* 8.91 (d, *J* = 8.4 Hz 1H, Ph-H), 8.21 (d, *J* = 9.2 Hz, 1H, Ph-H), 7.90 (m, 1H, Ph-H), 7.77 (d, *J* = 8.8 Hz, 2H, Ph-H), 7.60 (t, *J* = 8.0 Hz, 1H, Ph-H), 7.49 (d, *J* = 16.0 Hz, 1H, alkene-H), 7.36 (d, *J* = 16.0 Hz, 1H, alkene-H), 7.01 (s, 1H, C=CH), 6.82 (d, *J* = 8.8 Hz, 2H, Ph-H), 4.71 (t, *J* = 8.0 Hz, 2H, NC*H_2_*CH_2_CH_2_), 2.70 (t, *J* = 6.4 Hz, 2H, NCH_2_CH_2_C*H_2_*), 2.13 (m, 2H, NCH_2_C*H_2_*CH_2_). ^13^C NMR (100 MHz, DMSO-*d*_6_, ppm): *δ* 24.47, 45.89, 47.34, 47.58, 105.95, 115.64, 116.71, 118.36, 120.57, 124.80, 126.36, 130.50, 133.50, 138.09, 140.39, 149.79, 151.86, 159.46. High-resolution mass spectrometry (ESI negative ion mode for [M]^-^): Calcd. For C_23_H_18_N_3_O_4_S_1_: 432.1024; found: 432.1032.

1.7 Synthesis of QM-SO_3_-ER

QM-SO_3_-OH (92.0 mg, 0.2 mmol) and *N*-(2-chloroethyl)-4-methylbenzenesulfonamide (98.1 mg, 0.4 mmol) were dissolved in DMF (5.0 mL) with K_2_CO_3_ (116.0 mg, 0.8 mmol), under argon protection at room temperature, followed by heating at 45 °C for 12 h. The reaction solution was gradually poured into the 5% HCl solution (100.0 mL) under stirring to precipitate a red solid, which was then filtered with a funnel. The residue was separated by silica gel column chromatography, obtained the expected compound QM-SO_3_-ER (10.7 mg): yield: 8.12%. ^1^H NMR (400 MHz, DMSO-*d_6_*, ppm): *δ* 8.95 (t, *J* = 7.6 Hz, 1H, Ph-H), 8.23 (d, *J* = 8.8 Hz, 1H, Ph-H), 7.89 (m, 4H, Ph-H), 7.71 (d, *J* = 8.4 Hz, 2H, Ph-H), 7.49 (m, 5H, 2 for alkene-H, 1 for NH, 4 for Ph-H), 7.07 (s, 1H, C=CH), 6.89 (d, *J* = 8.8 Hz, 2H, Ph-H), 4.74 (t, *J* = 7.2 Hz, 2H, NC*H_2_*CH_2_CH_2_), 4.02 (d, *J* = 5.6 Hz, 2H, O*CH_2_*CH_2_), 2.09 (d, *J* = 3.2 Hz 2H, NCH_2_CH_2_C*H_2_*), 2.70 (d, *J* = 5.6 Hz, 2H, OCH_2_C*H_2_*), 2.38 (s, 3H, CH_3_), 2.13 (s, 2H, NCH_2_C*H_2_*CH_2_). ^13^C NMR (100 MHz, DMSO-*d_6_*, ppm): *δ* 24.47, 45.89, 47.34, 47.58, 105.95, 115.64, 116.71, 118.36, 120.57, 124.80, 126.36, 130.50, 133.50, 138.09, 140.39, 149.79, 151.86, 159.46. High-resolution mass spectrometry (ESI negative ion mode for [M]^-^): Calcd. For C_32_H_29_N_4_O_6_S_2_: 629.1354; found: 629.1534.

1.8 Preparation of liposomes

The egg yolk lecithin was dissolved in chloroform at concentration of 10 mg mL^-1^, then the chloroform was dried under a stream of nitrogen to obtain lipid film. The film was hydrated by incubating with HEPES buffer (25 mM, pH 7.4) at 37 ℃ for 30 minutes to a final lipid concentration of 2.2 mM. The rough liposomes were harvested, sonicated for 1 h, and extruded 11 times through 100 nm pore size polycarbonate filter at 50 °C on a pre-warmed lipid extruder, so as to obtain the final liposomes. The critical micelle concentration (CMC) of egg yolk lecithin is extremely low (1.51 mM) and spontaneously form double vesicles easily. In this work, the final concentration of egg yolk lecithin (2.2 mM) is higher than the CMC to success form liposomes [33,43].

1.9 **Partition-coefficient (Log*P*) measurement**

LogP was determined by shake-flask method. Water and octanol were mixed and fully shook to equilibrant, then the two layers were placed to separate. The AIE probe was dissolved in 1 mL of water saturated octanol, followed by adding 9 mL octanol saturated water into it. The mixture was shook vigorously at 37 ℃ for 24 h. Then the concentration of AIE probe was determined by UV-vis spectroscopy and calculated using the following formula. QM-SO_3_-ER of Log*P*_o/w_ 1.61 is more hydrophilic than QM-ER of 1.23.

**LogP o/w = log (**$\frac{\mathbf{[solute]}\mathbf{octanol}}{\mathbf{[solute]}\mathbf{water}}\mathbf{)}$

1.10 Molecular Docking Assay

The molecular structure was constructed using the Discovery Studio 2016 software, based on an X-ray crystal structure (PDB ID: 6BAA). The input files for the calculations were generated with the Discovery Studio Visualizer client, and docking calculations were carried out using DS LibDock Modual in Discovery Studio 2016 software. Visualization of the calculation results was performed using the Discovery Studio Visualizer client.

1.11 Cell lines

The human hepatocyte cell (QSG-7701), adenocarcinomic human alveolar basal epithelial cells (A549), human epithelioid cervical carcinoma cell (HeLa) and human pancreatic cancer cell (PANC-1) were purchased from the Institute of Cell Biology (Shanghai, China). Cells were all propagated in T-75 flasks cultured at 37 ℃ under a humidified 5% CO_2_ atmosphere in DMEM medium (GIBCO/Invitrogen, Camarillo, CA, USA), which were supplemented with 10% fetal bovine serum (FBS, Biological Industry, Kibbutz Beit Haemek, Israel) and 1% penicillin-streptomycin (10,000 U mL^-1^ penicillin and 10 mg mL^-1^ streptomycin, Solarbio life science, Beijing, China).

**1.12 *In vitro* cytotoxicity assay**

The cell cytotoxicity of QM-ER and QM-SO_3_-ER in HeLa cells was evaluated by a MTT (3-(4,5-dimethylthiazol-2-yl)-2,5-diphenyltetrazolium bromide) assay. Briefly, cells were seeded into 96-well plates at a density of 1 × 10^4^ cells/well and were cultured at 37 ºC under a humidified 5% CO_2_ atmosphere for 12 h. Then, the cells were exposed to the various concentrations (1, 2.5, 5.0, 7.5, 10, 15 μM) of QM-ER and QM-SO_3_-ER, and for negative control group, 100 μL of culture medium were added. After incubation at 37 ºC under a humidified 5% CO_2_ atmosphere for 24 h, MTT solution (5 mg/mL in PBS, 10 μL) was added to the media and incubated for another 4 h, and the absorbance at 490 nm was measured with a Multimode Plate Reader (BioTek, USA). The relative cell viability (%) was calculated by the following formula: Cell viability (%) = mean absorbance value of the treatment group-blank/mean absorbance value of the control blank × 100%.

1.13 Cells imaging

HeLa cells were seeded onto glass-bottom Petri dishes in culture medium (1.0 mL) and allowed to adhere for 12 h before imaging. Probe QM-ER and QM-SO_3_-ER at a final concentration of 3 × 10^-6^ M (containing 0.1% DMSO) were added into culture medium and incubated for different time at 37 °C under a humidified 5% CO_2_ atmosphere. Cells imaging was captured by using a confocal laser scanning microscope (CLSM, Nikon A1R system, Japan) with a 60 × oil immersion objective lens. The fluorescence signals of cells incubated with probes were collected at 550-630 nm under excitation wavelength at 405 nm.

2. Absorption and emission spectra of QM-OH and QM-SO_3_-OH

**
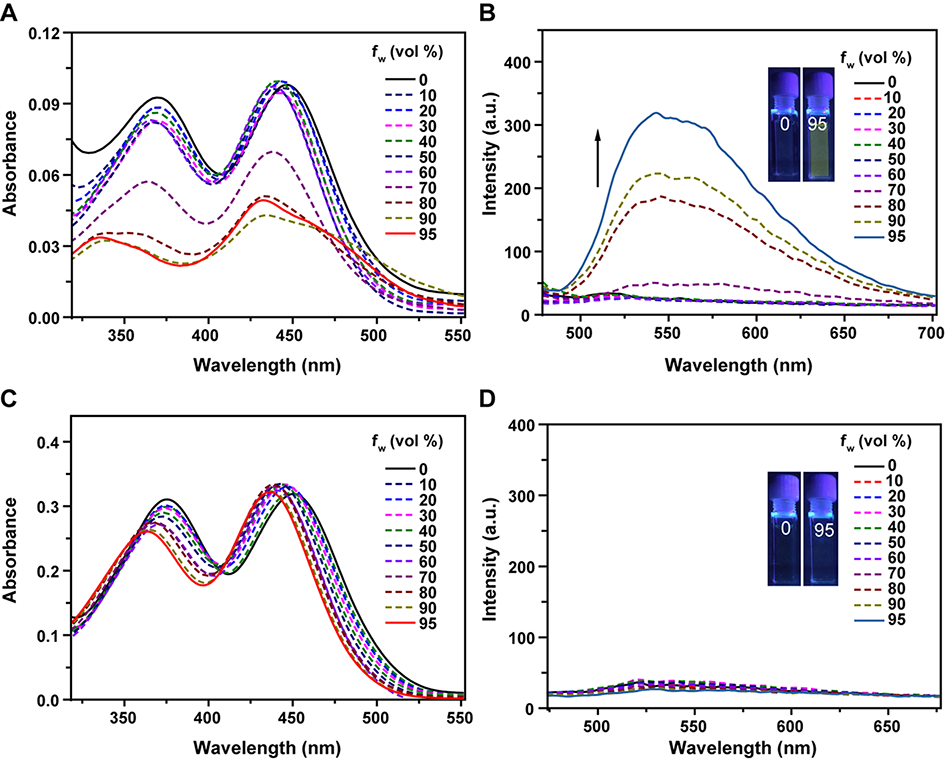
**

**Figure S1.** Absorption and emission spectra of QM-OH and QM-SO_3_-OH in THF-Water. (A and C) Absorption spectra of QM-OH and QM-SO_3_-OH in a mixture of THF-Water with different water fractions (*f*_w_), respectively. (B and D) Emission spectra of QM-OH and QM-SO_3_-OH in a mixture of THF-Water with different water fractions (*f*_w_), respectively. Inset: images of QM-OH and QM-SO_3_-OH with various *f*_w_ under UV lamp illumination.

**3. ACQ effect of ER-tracker Red**


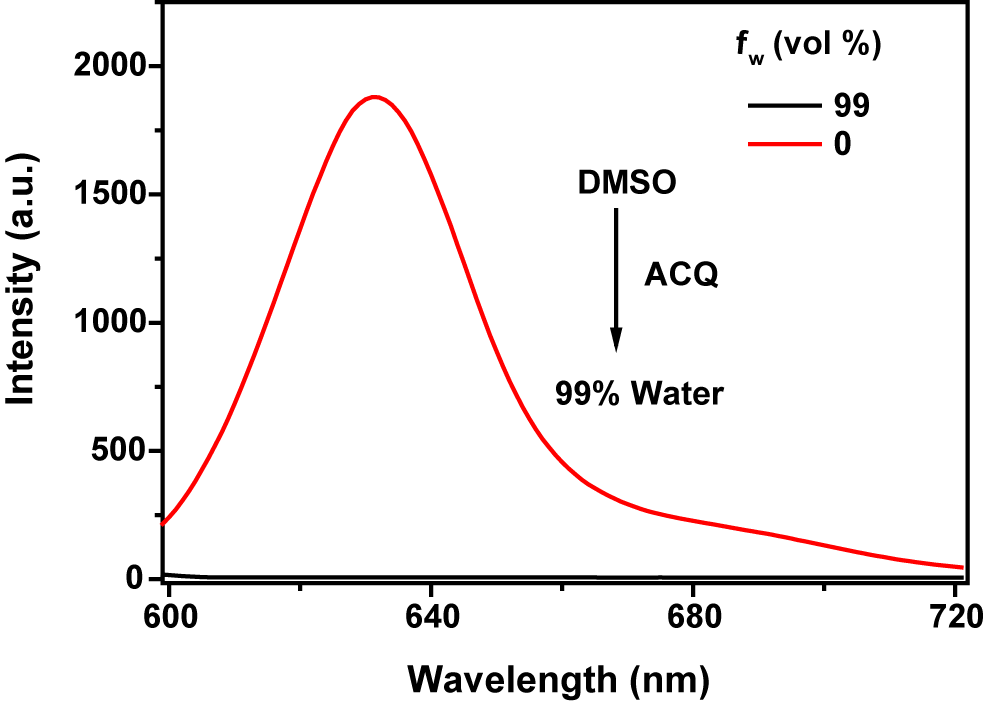


**Figure S2.** The emission spectra of ER-tracker Red (10 μM) in water and DMSO, indicative of the typical aggregation caused quenching (ACQ) effect of ER-tracker Red.

4. TEM images of QM-ER and **QM-SO_3_-ER**

**
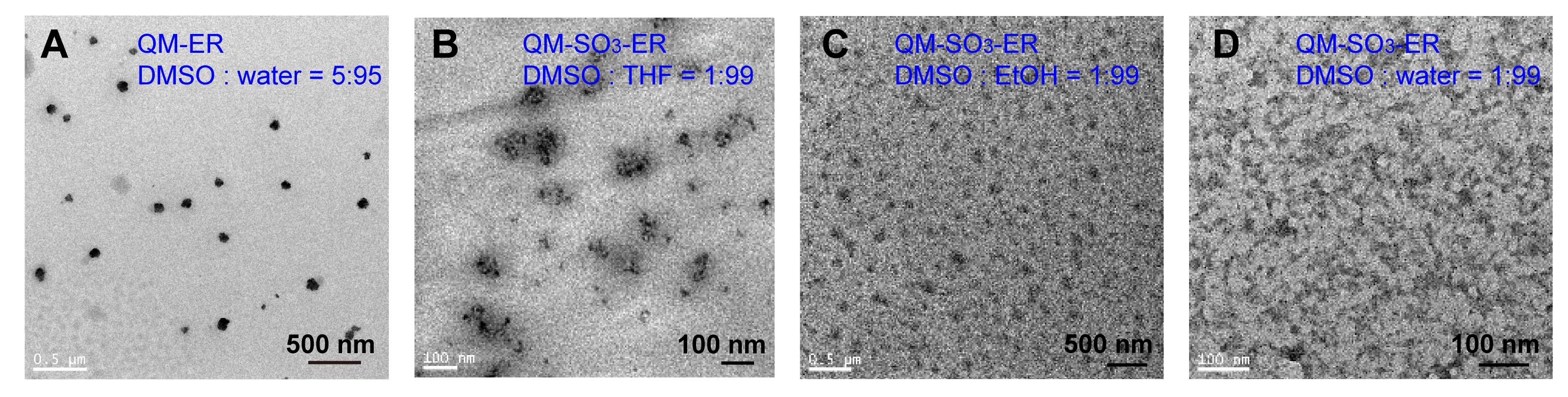
**

**Figure S3.** (A) TEM image of QM-ER aggregates (10 μM) in a mixture of DMSO/water (v/v = 5/95). (B-D) TEM images of QM-SO_3_-ER aggregates (10 μM) in different solvent system: (A) DMSO/THF (v/v = 1/99), (B) DMSO/EtOH (v/v = 1/99), (C) DMSO/water (v/v = 1/99).

5. Absorption spectra of QM-ER and QM-SO_3_-ER in various systems


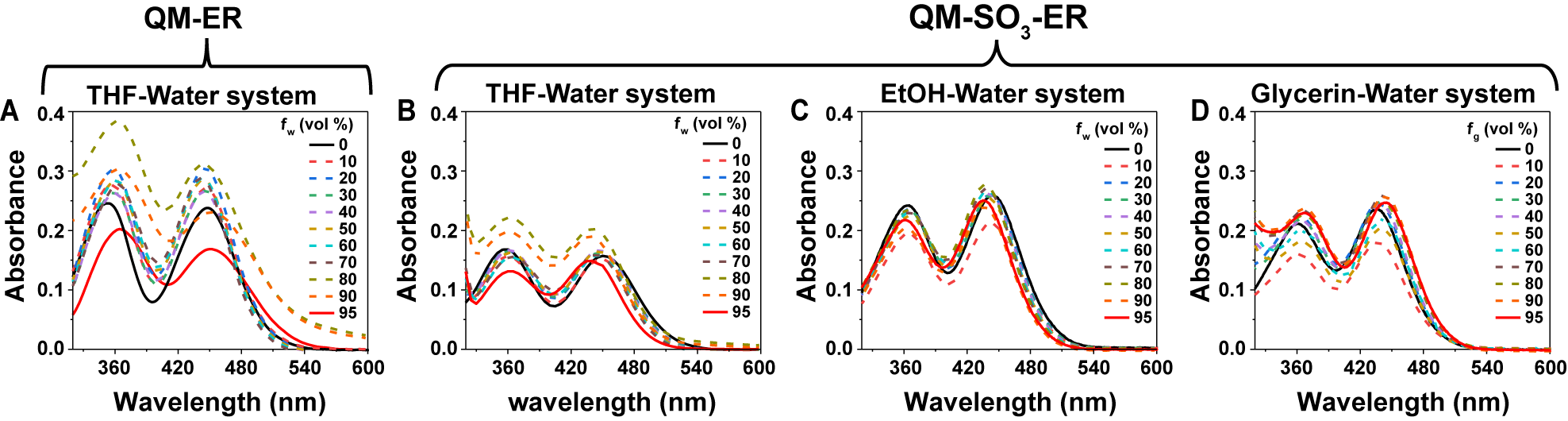


**Figure S4.** Absorption spectra of QM-ER and QM-SO_3_-ER with different fraction of water. (A) Absorption spectra of QM-ER in THF- Water system with different volume fractions of water (*f*_w_). (B-D) Absorption spectra of QM-SO_3_-ER (10 μM) in THF-Water, EtOH-Water, and Glycerin-Water, respectively.

6. Absorption and emission spectra of QM-SO_3_-ER in DMSO-water systems

**
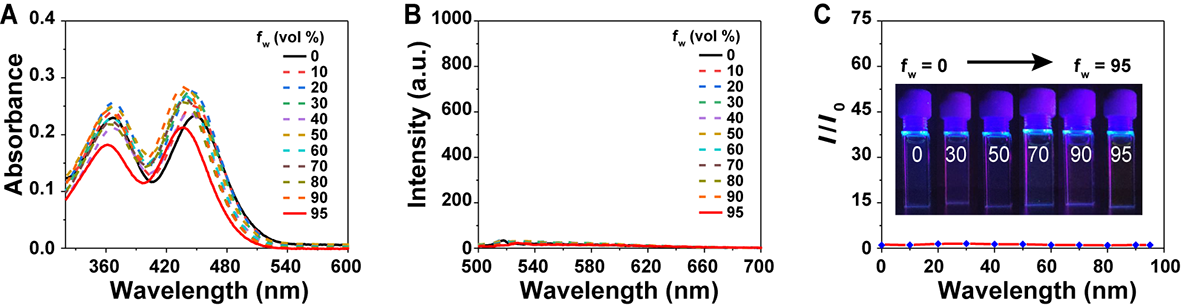
**

**Figure S5.** Spectral properties of QM-SO_3_-ER with different water fractions (*f*_w_) in DMSO-Water system. (A and B) Absorption and emission spectra of QM-SO_3_-ER in DMSO-Water systems, *λ*_ex_ = 447 nm. (C) *I/I_0_* plots of QM-SO_3_-ER (10 μM), where *I* is the fluorescence intensity of fluorophore in 95% water at 589 nm and *I_0_* is the fluorescence intensity of fluorophore in 0% water, *λ*_ex_ = 447 nm.

7. Emission spectra of QM-SO_3_-ER in liposomes


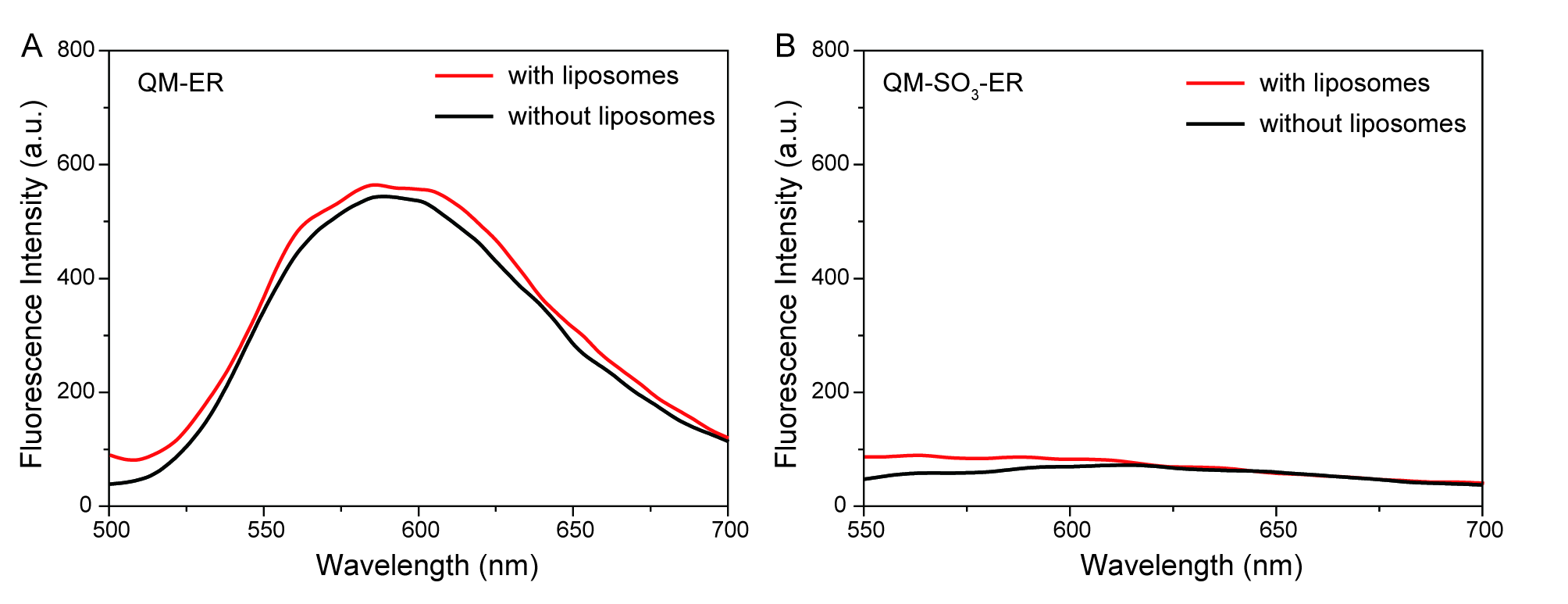


**Figure S6.** Fluorescence spectra of QM-ER (1 × 10^−5^ mol L^-1^) (A) and QM-SO_3_-ER (1 × 10^−5^ mol L^-1^) (B) in the presence of liposomes (4 × 10^−5^ mol L^-1^) in PBS buffer solution, *λ*_ex_ = 447 nm. In contrast with QM-ER, the *amphiphilic* QM-SO_3_-ER senor could overcome the unexpected aggregation in bio-application.

**8. Hydrodynamic diameter of QM-SO_3_-ER**


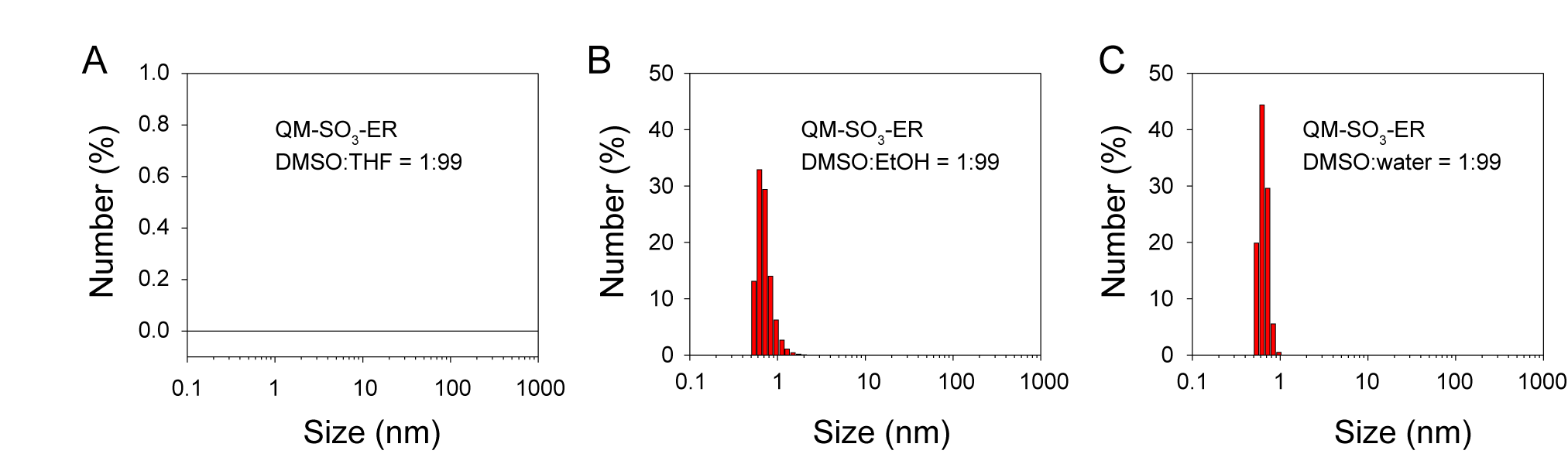


**Figure S7.** Hydrodynamic diameter of QM-SO_3_-ER (10 μM) in a mixture of (A) DMSO/THF (v/v = 1/99), (B) DMSO/EtOH (v/v = 1/99) and (C) DMSO/water (v/v = 1/99) obtained from dynamic light scattering (DLS), indicative of the good solubility in a mixture of DMSO/THF.

9. Solid state fluorescence spectra of QM-ER and QM-SO_3_-ER


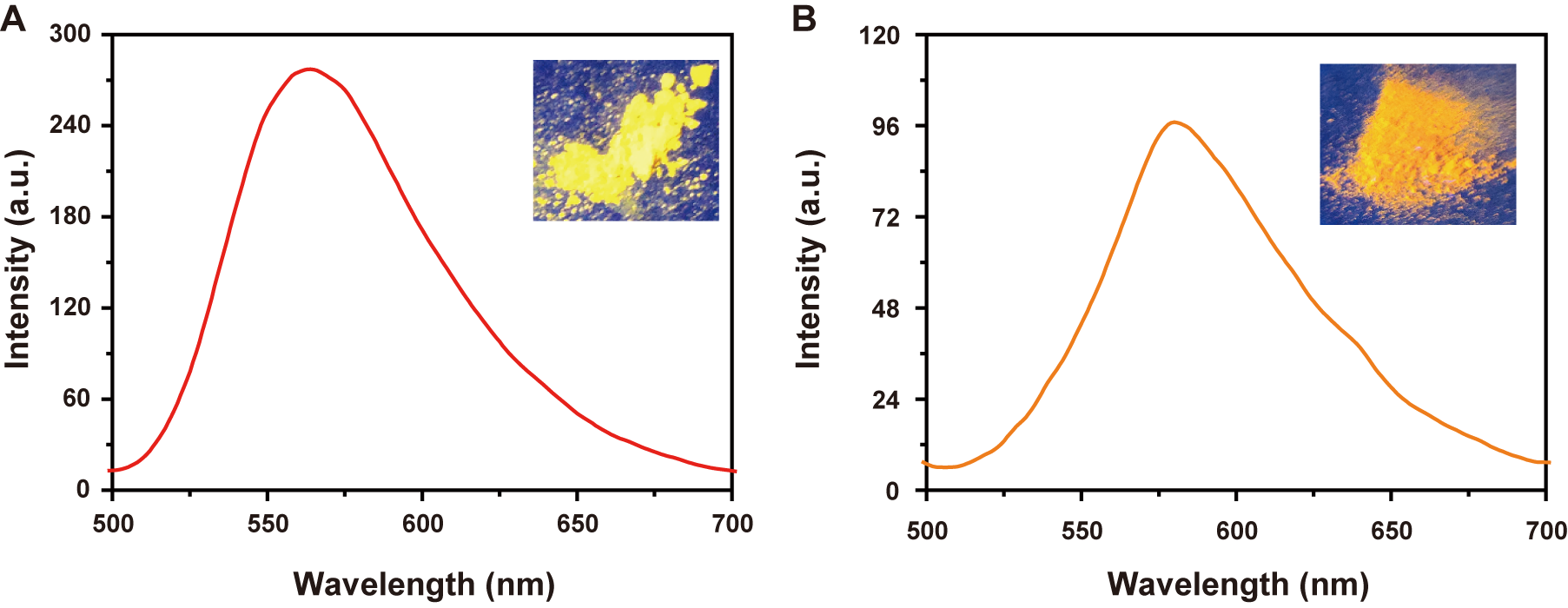


**Figure S8.** Solid state fluorescence spectra of QM-ER and QM-SO_3_-ER. Fluorescent spectra of (A) QM-ER and (B) QM-SO_3_-ER in the solid state, *λ*_ex_ = 450 nm. Insert: the fluorescence photographs of compounds QM-ER and QM-SO_3_-ER, respectively.

10. Overall structure of the K_ATP_ channel bound QM-SO_3_-ER

**
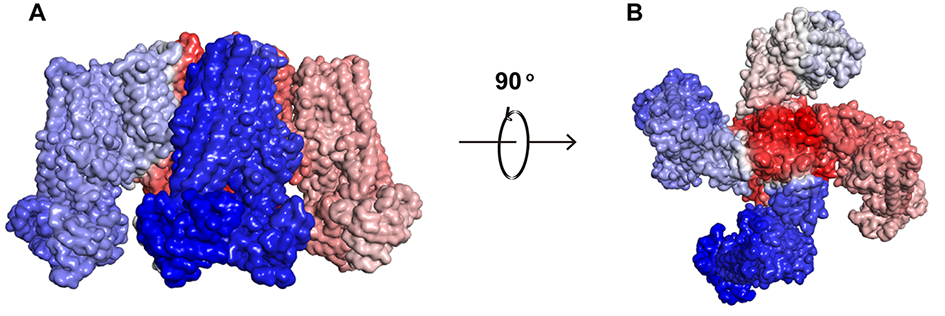
**

**Figure S9.** Overall structure of the K_ATP_ protein. (A) Cryo-EM density map of the K_ATP_ channel complex, viewed from the side. (B) Cryo-EM density map of the K_ATP_ channel complex, viewed from extracellular side.

**11. Co-localization experiment of QM-SO_3_-ER and ER-tracker Red**


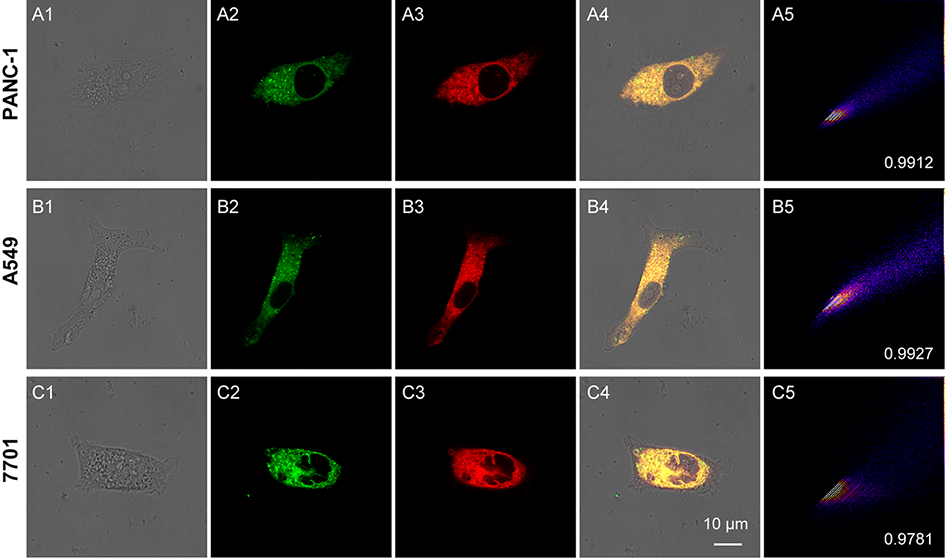


**Figure S10.** Co-localization experiment of QM-SO_3_-ER (3 μM) and ER-tracker Red (1 μM) in PANC-1, A549 and 7701 cells. Note: The QM-SO_3_-ER overlapped well with ER-tracker Red in these cell lines.

12. Photostability of ER-tracker Red, QM-ER and QM-SO_3_-ER *in vitro*

**
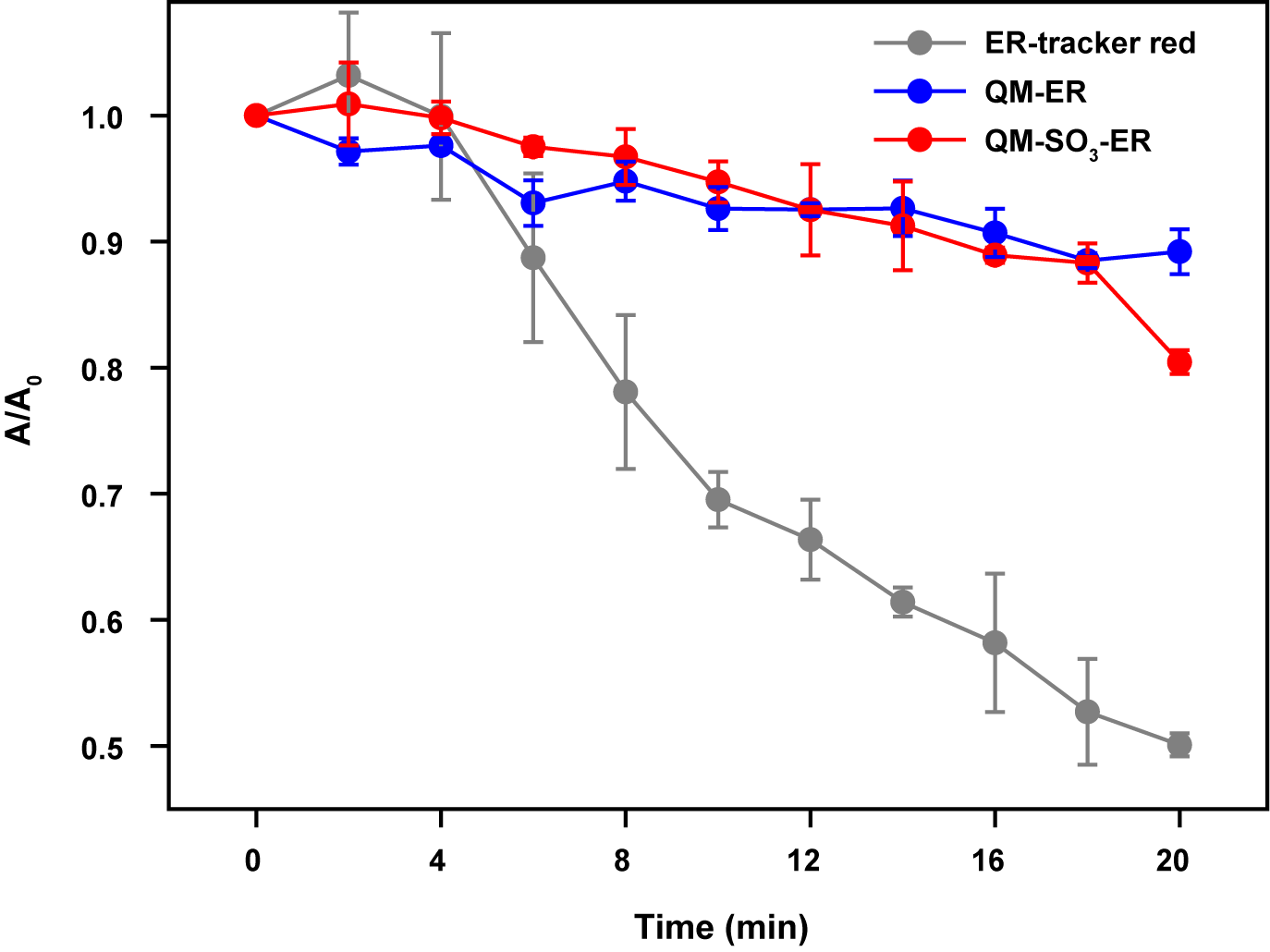
**

**Figure S11.** Time-dependent absorbance of ER-tracker Red (1 μM), QM-ER (1 μM) and QM-SO_3_-ER (1 μM) under sustained illumination.

**13. Characterization of compounds**


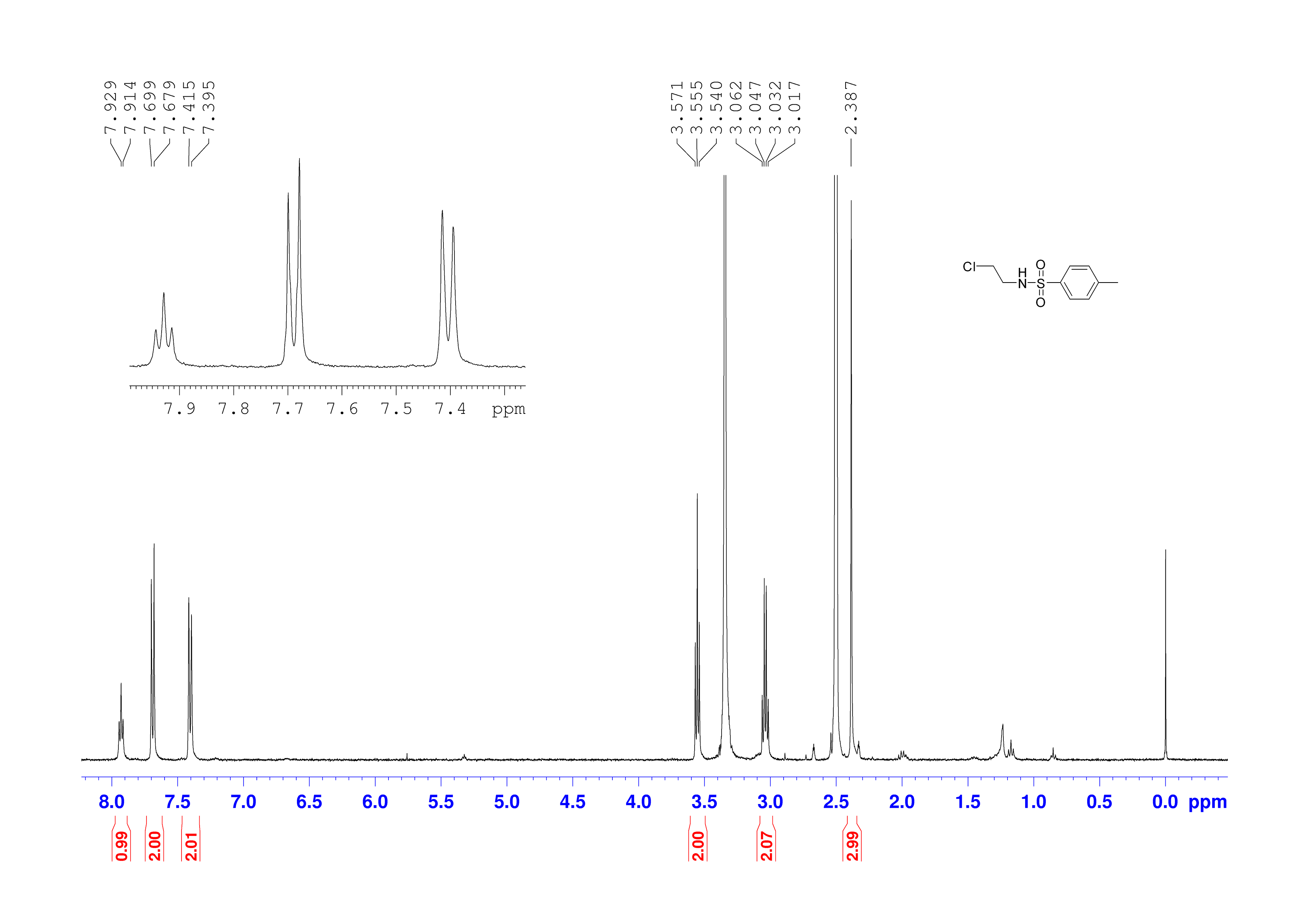


**Figure S12.** ^1^H NMR spectrum of ER-targeting moiety in DMSO-*d*_6。_


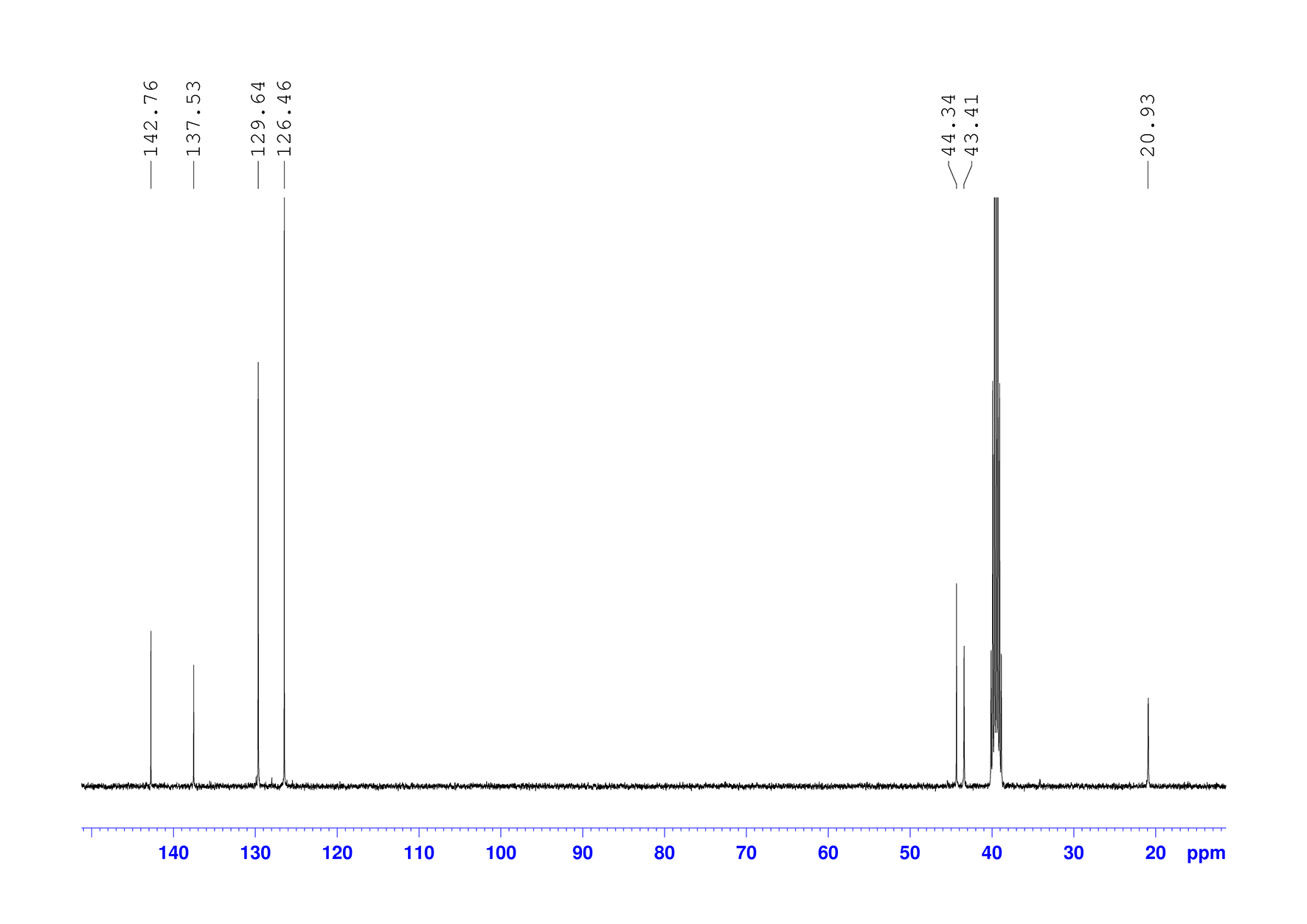


**Figure S13.** ^13^C NMR spectrum of ER-targeting moiety in DMSO-*d*_6_.


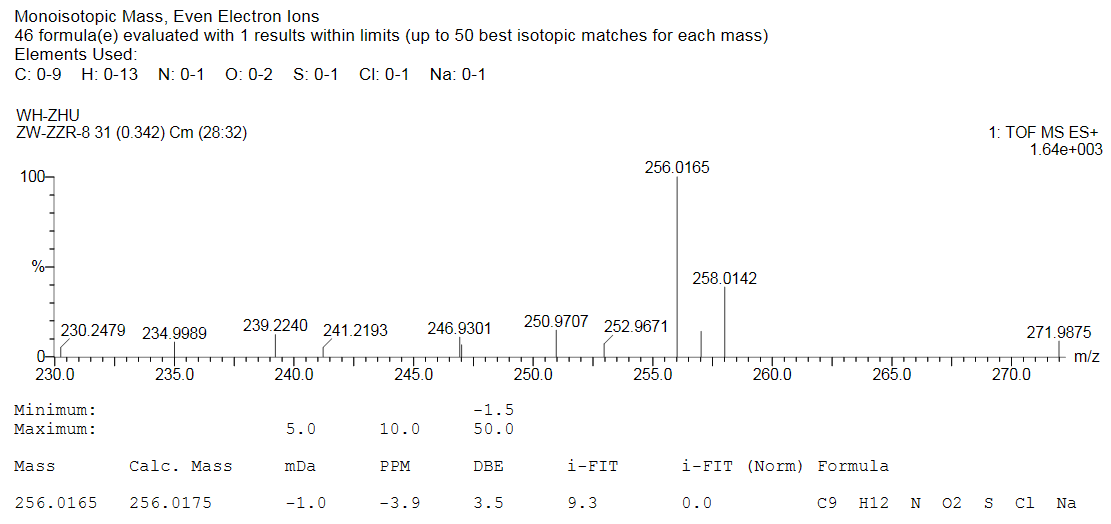


**Figure S14.** HRMS spectrum of ER-targeting moiety.


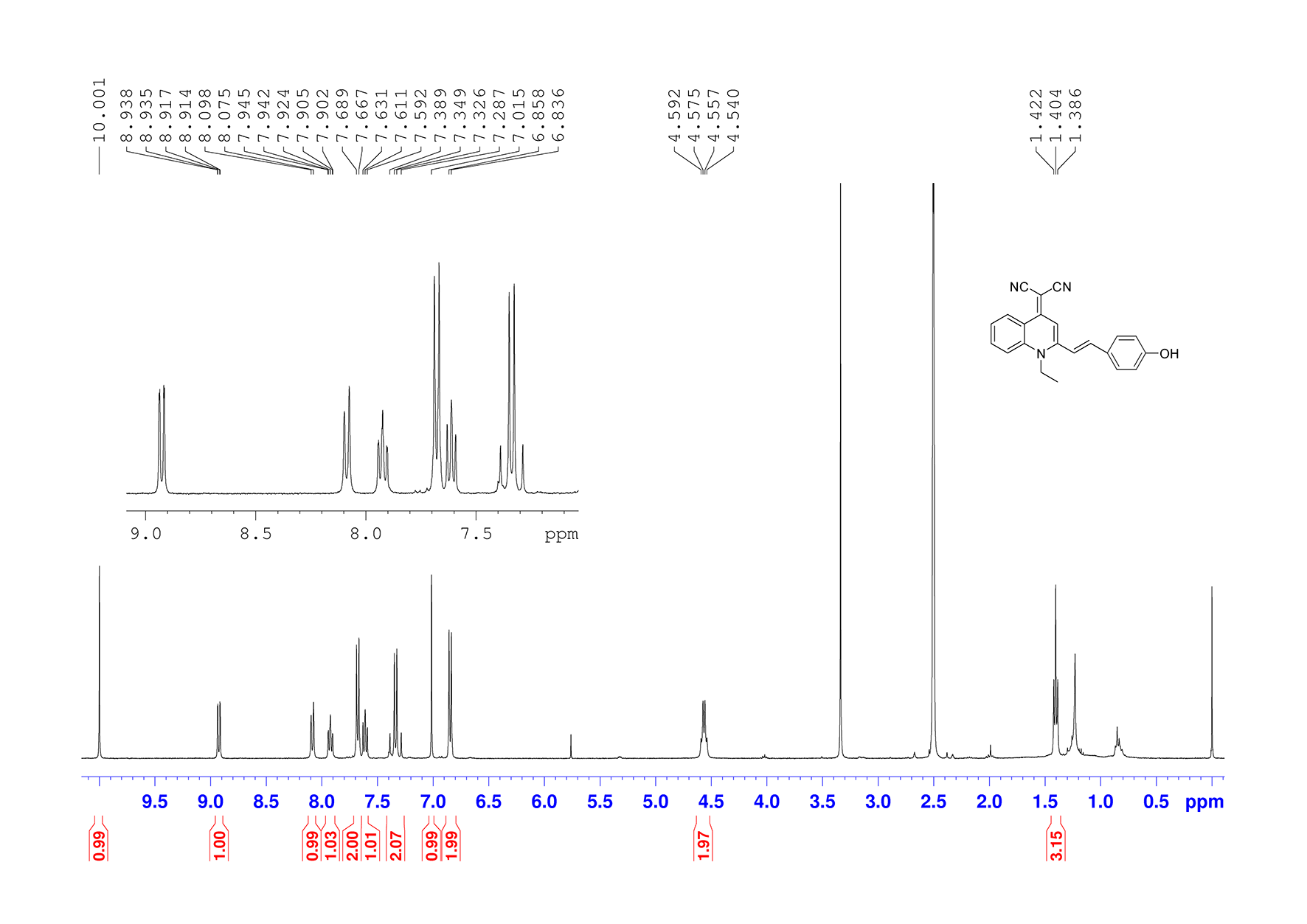


**Figure S15.** ^1^H NMR spectrum of QM-OH in DMSO-*d*_6_.


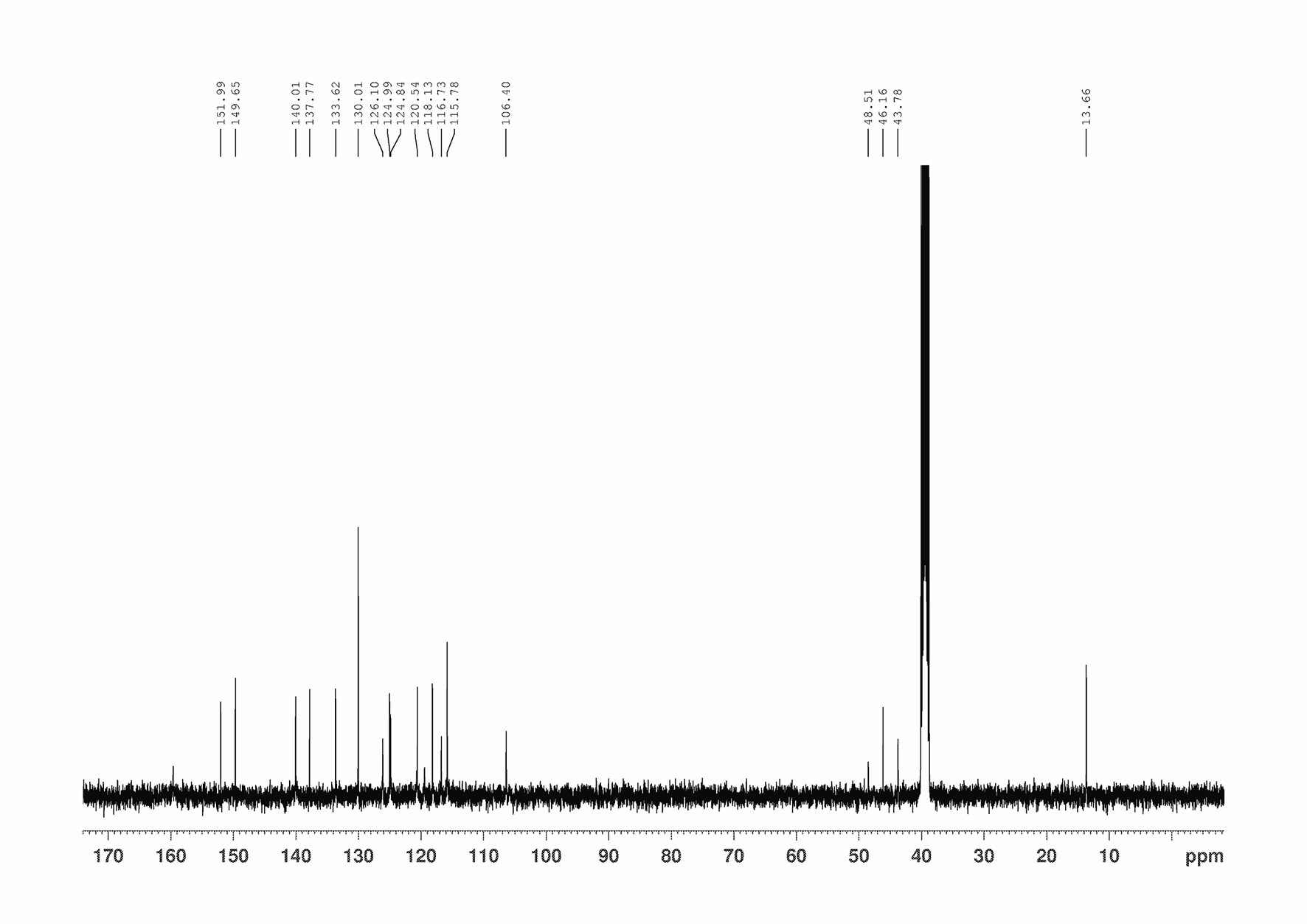


**Figure S16.** ^13^C NMR spectrum of QM-OH in DMSO-*d*_6_.


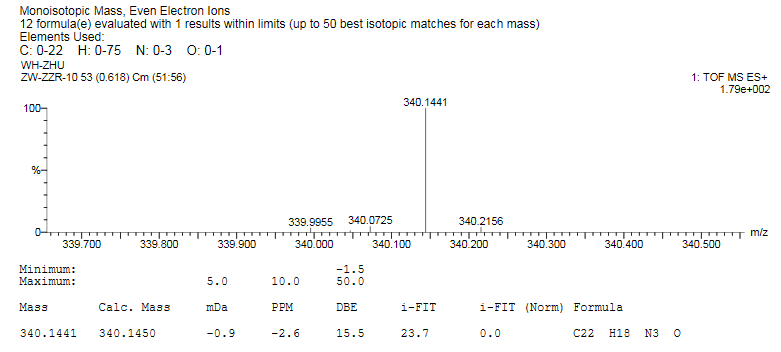


**Figure S17.** HRMS spectrum of QM-OH.


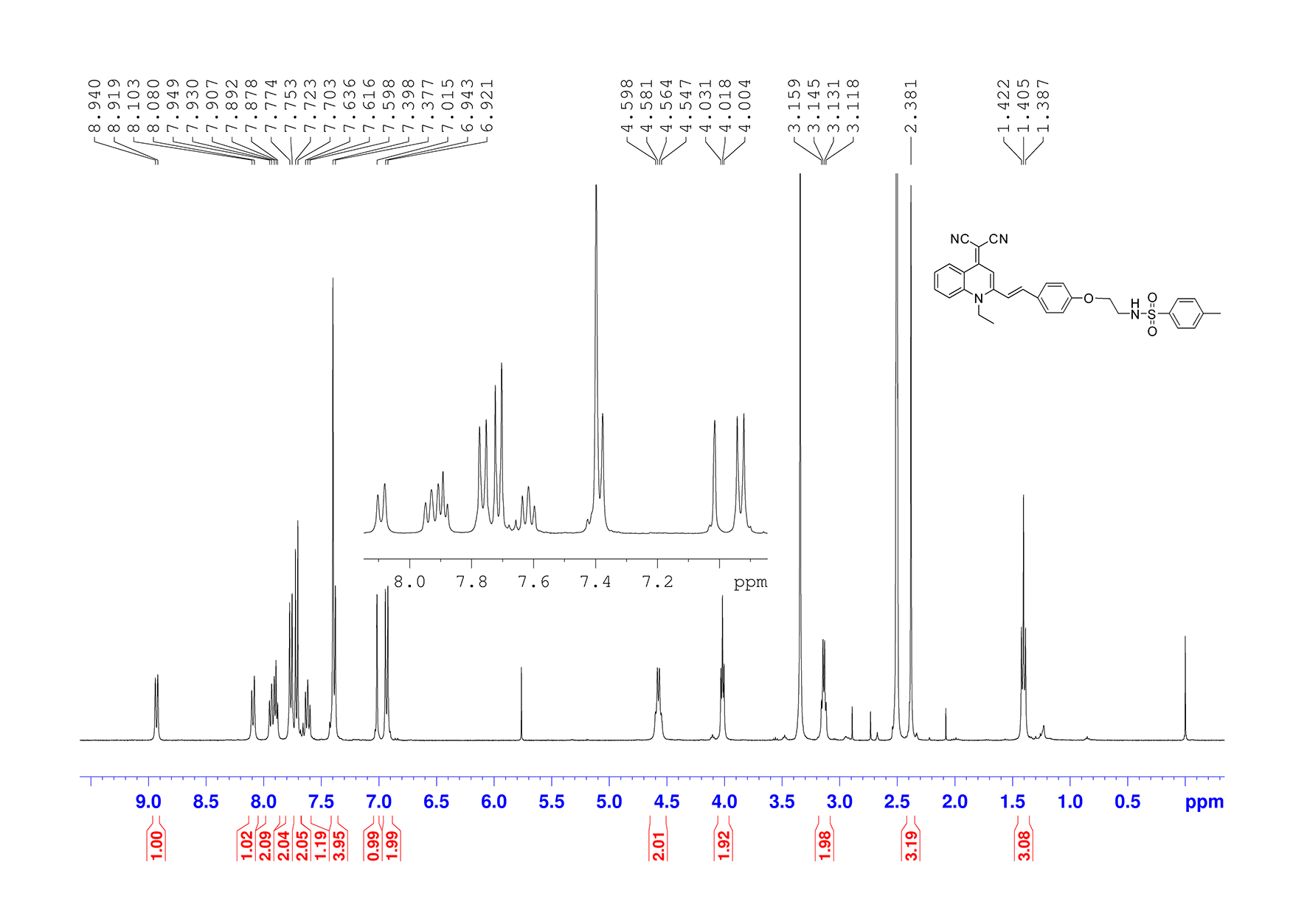


**Figure S18.** ^1^H NMR spectrum of QM-ER in DMSO-*d*_6_.


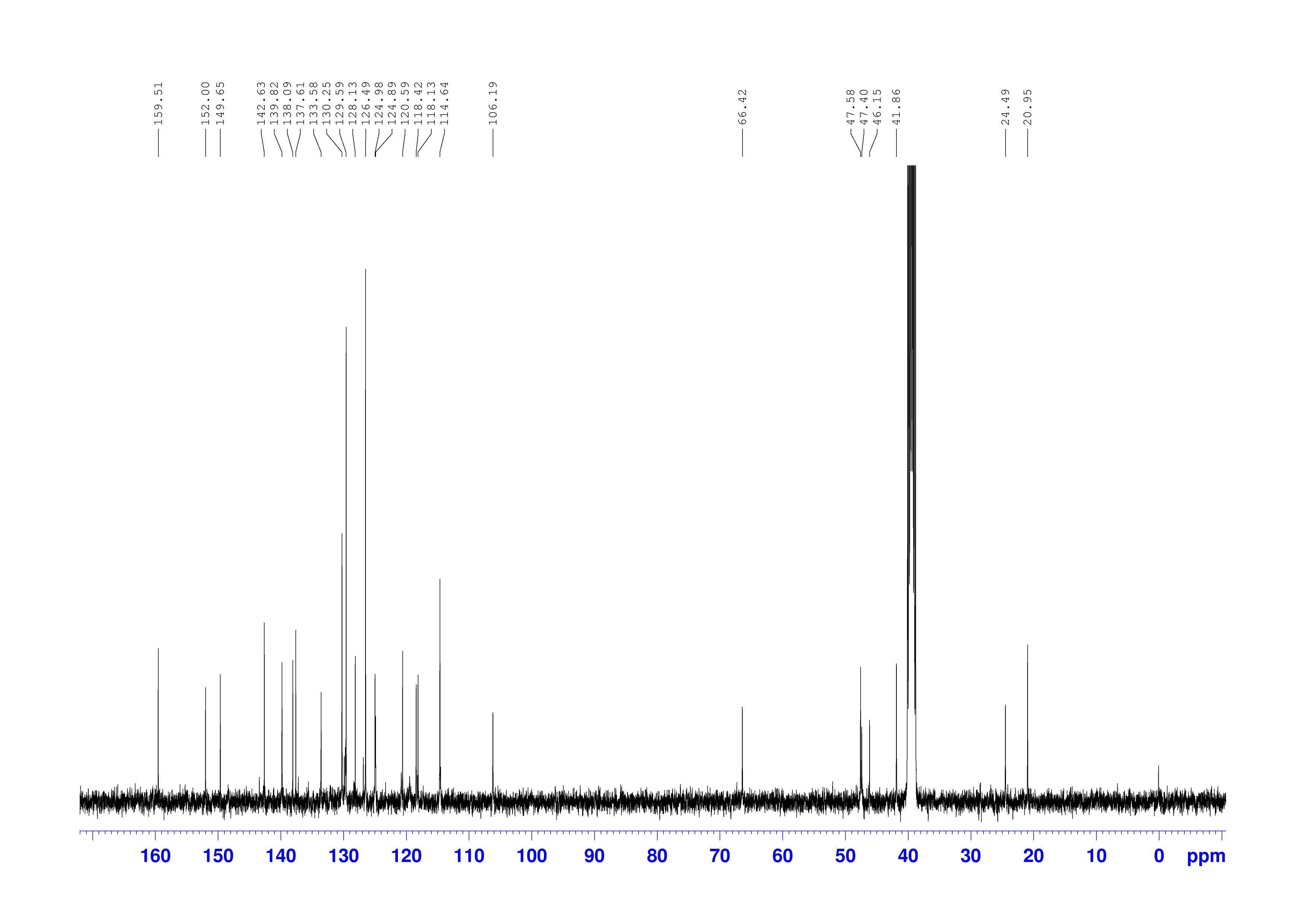


**Figure S19.** ^13^C NMR spectrum of QM-ER in DMSO-*d*_6_.


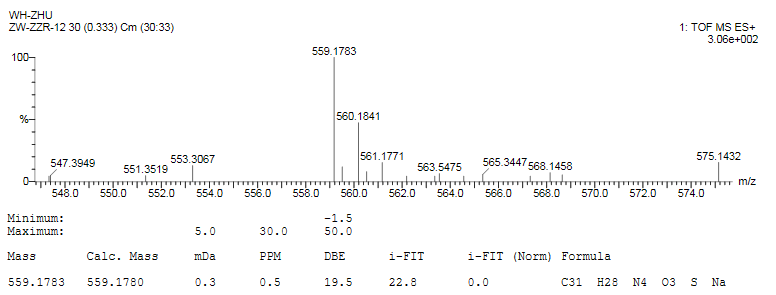


**Figure S20.** HRMS spectrum of QM-ER.


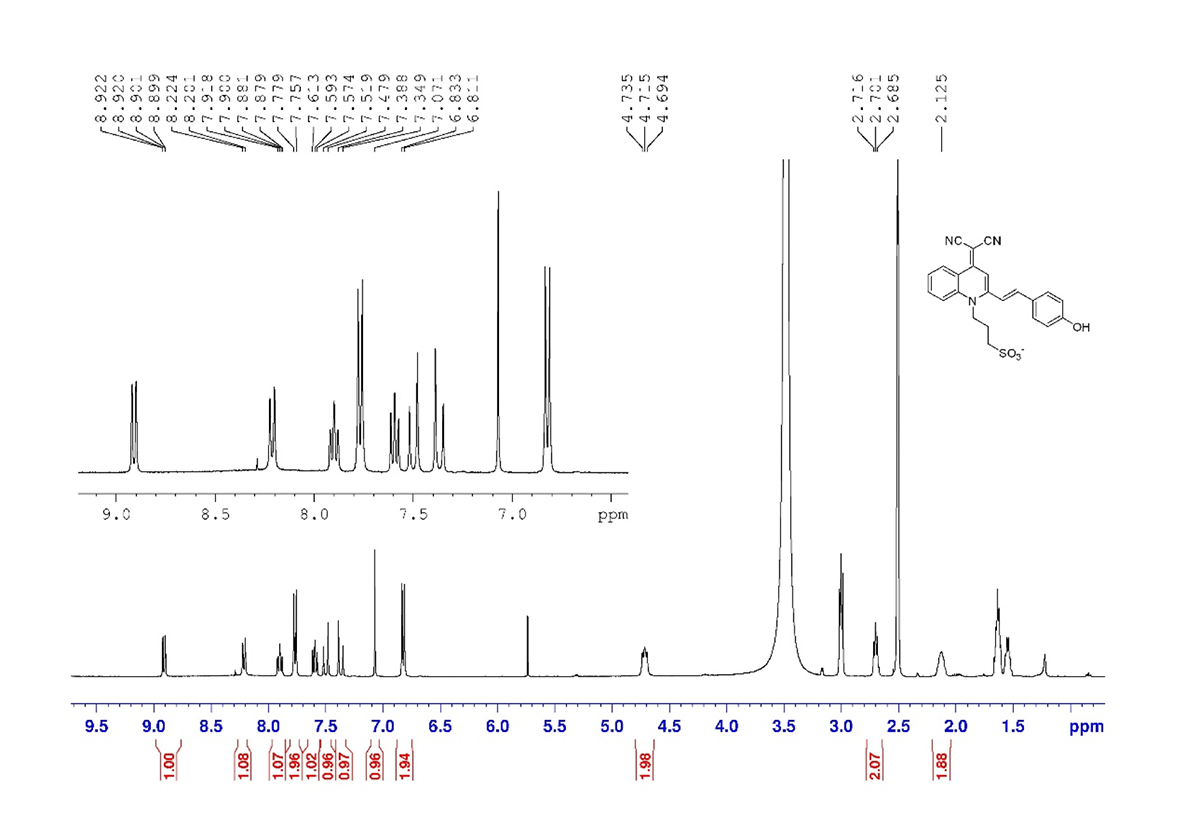


**Figure S21.** ^1^H NMR spectrum of QM-SO_3_-OH in DMSO-*d*_6_.


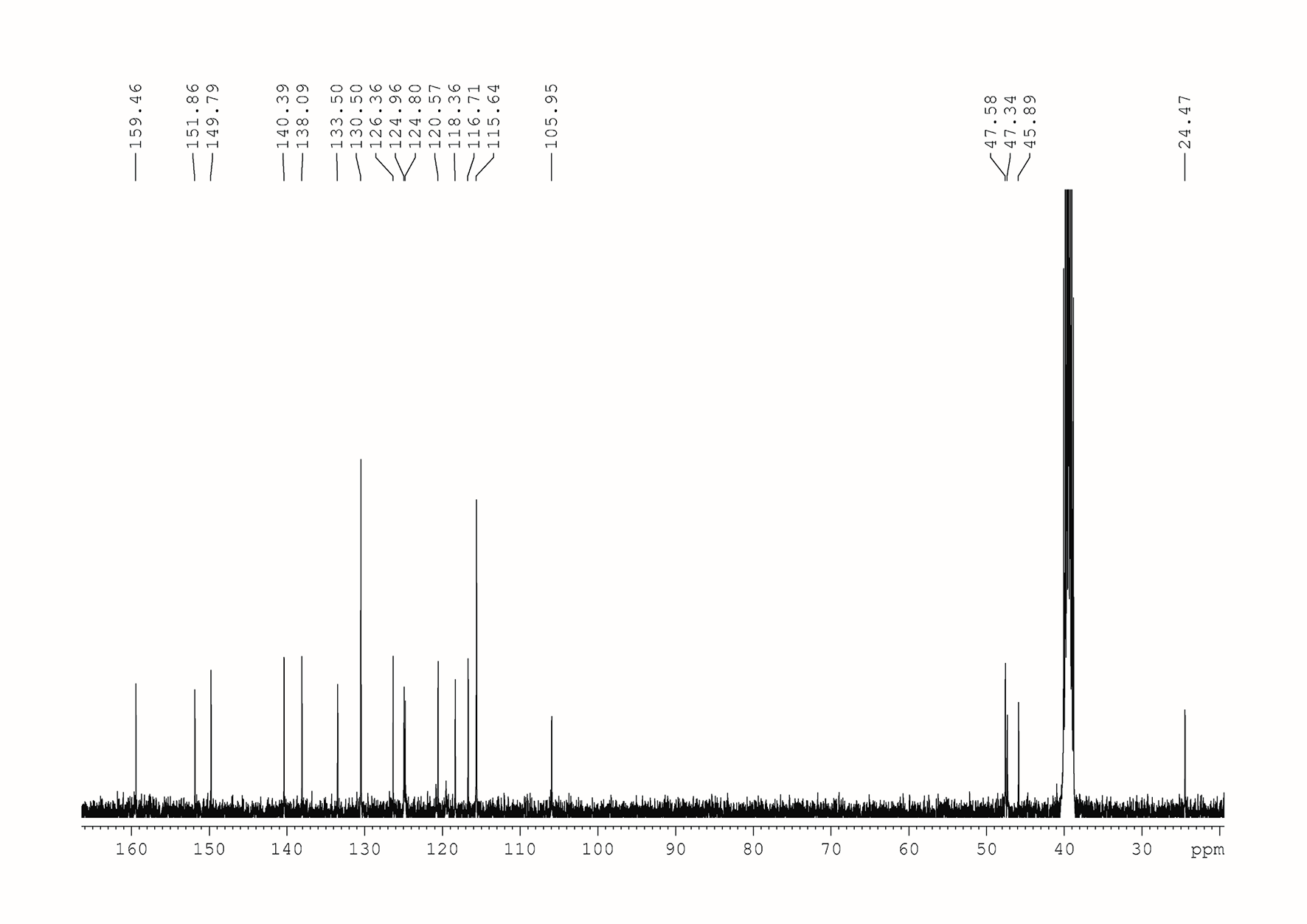


**Figure S22.** ^13^C NMR spectrum of QM-SO_3_-OH in DMSO-*d*_6._


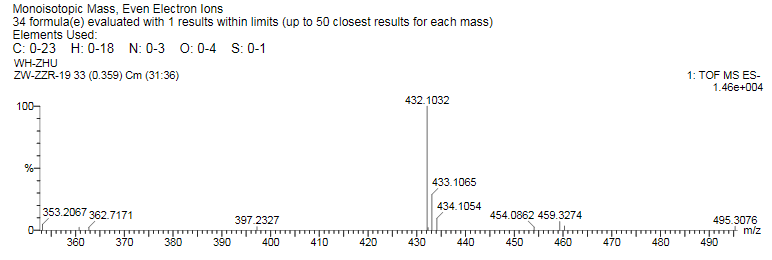


**Figure S23.** HRMS spectrum of QM-SO_3_-OH.


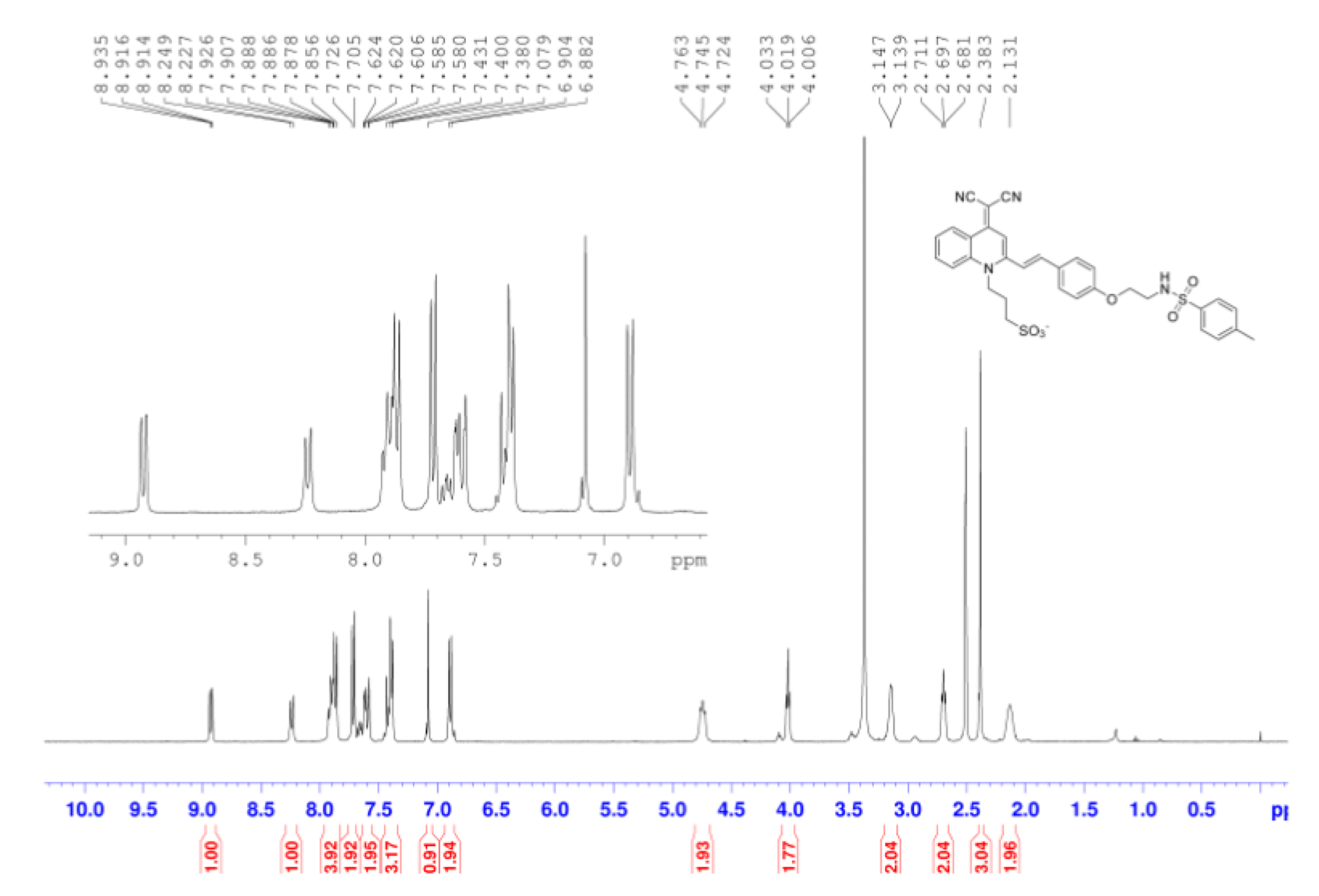


**Figure S24.** ^1^H NMR spectrum of QM-SO_3_-ER in DMSO-*d*_6_.


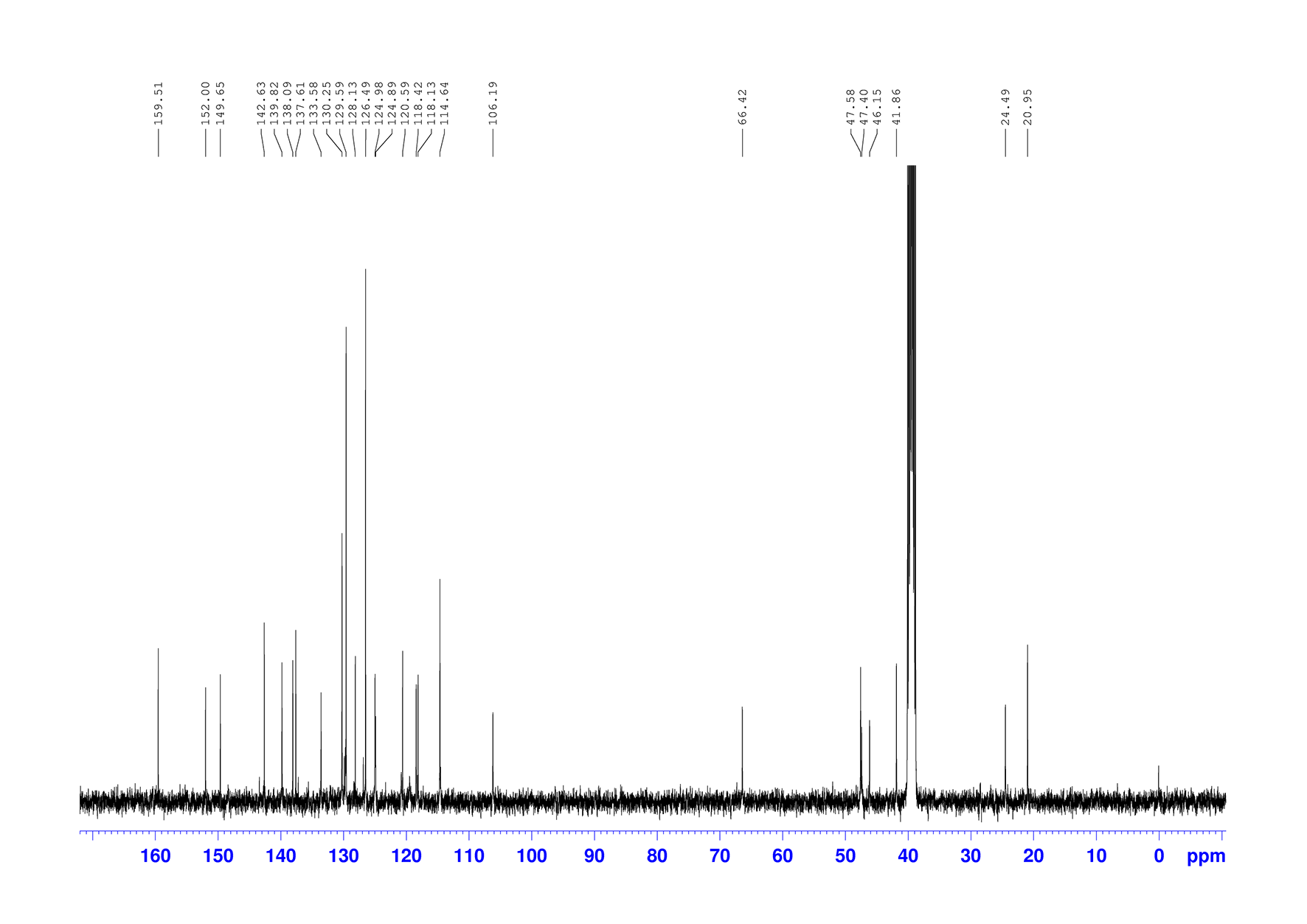


**Figure S25.** ^13^C NMR spectrum of QM-SO_3_-ER in DMSO-*d*_6._


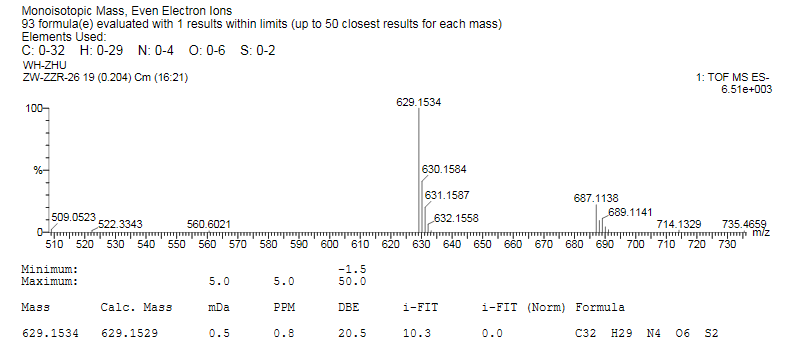


**Figure S26.** HRMS spectrum of QM-SO_3_-ER.
